# Eyes on Prevention: An Eye-Tracking Analysis of Visual Attention Patterns in Breast Cancer Screening Ads

**DOI:** 10.3390/jemr18060075

**Published:** 2025-12-13

**Authors:** Stefanos Balaskas, Ioanna Yfantidou, Dimitra Skandali

**Affiliations:** 1 Department of Physics, School of Sciences, Democritus University of Thrace, Kavala Campus, 65404 Kavala, Greece; 2 Department of Business and Management, Liverpool John Moores University (LJMU), Rodney Street, Liverpool L3 5UL, UK; i.yfantidou@ljmu.ac.uk; 3 Department of Business Administration, National and Kapodistrian University of Athens, 10679 Athens, Greece; dskandali@ba.uoa.gr

**Keywords:** eye-tracking, visual attention, breast cancer screening, public service advertising, health communication, message design, generalized linear mixed models (GLMMs), scan–then–read

## Abstract

Strong communication is central to the translation of breast cancer screening availability into uptake. This experiment tests the role of design features of screening advertisements in directing visual attention in screening-eligible women (≥40 years). To this end, a within-subjects eye-tracking experiment (N = 30) was conducted in which women viewed six static public service advertisements. Predefined Areas of Interest (AOIs), Text, Image/Visual, Symbol, Logo, Website/CTA, and Source/Authority—were annotated, and three standard measures were calculated: Time to First Fixation (TTFF), Fixation Count (FC), and Fixation Duration (FD). Analyses combined descriptive summaries with subgroup analyses using nonparametric methods and generalized linear mixed models (GLMMs) employing participant-level random intercepts. Within each category of stimuli, detected differences were small in magnitude yet trended towards few revisits in each category for the FC mode; TTFF and FD showed no significant differences across categories. Viewing data from the perspective of Areas of Interest (AOIs) highlighted pronounced individual differences. Narratives/efficacy text and dense icon/text callouts prolonged processing times, although institutional logos and abstract/anatomical symbols generally received brief treatment except when coupled with action-oriented communication triggers. TTFF timing also tended toward individual areas of interest aligned with the Scan-Then-Read strategy, in which smaller labels/sources/CTAs are exploited first in comparison with larger headlines/statistical text. Practically, screening messages should co-locate access and credibility information in early-attention areas and employ brief, fluent efficacy text to hold gaze. The study adds PSA-specific eye-tracking evidence for breast cancer screening and provides immediately testable design recommendations for programs in Greece and the EU.

## 1. Introduction

Breast cancer is still one of the most significant public health problems and the leading cancer in women worldwide. In 2022, a total of 2.3 million women were diagnosed with breast cancer, and 670.000 deaths occurred due to it [1,2]. It is a big difference if treated and detected early on—death is significantly minimized if breast cancer is diagnosed in its early stages. Mammography screening is therefore highly recommended for midlife women (typically 50–69 years) as an evidence-based therapy to promote survival [1]. In the past two decades, there has been the implementation of organized breast cancer screening programs in Europe and the improvement of coverage on a step-by-step basis. In the EU, on average, around 56% of invited women now receive breast screening according to recent figures. However, with highly varied participation between countries, the Nordic countries reach over 80% take-up [1,2], while others fall well below. For example, Greece has also registered one of the lowest mammography screening rates in the European Union, with only approximately 14–15% of women who are due for a mammogram within the appropriate time having taken one as of 2023 [1,2]. These disparities in screening coverage then affect early detection and trends of cancer mortality, illustrating the necessity of identifying and bridging the barriers to screening uptake.

In Greece, rising attendance for screening has been made a national priority. Up to the present date, Greek breast screening continued to be opportunistic—women achieved mammograms on an individual or doctor-initiated basis—and based on available survey data, around 66% of Greek women aged 50–69 years had received a mammogram within the past two years (up to 2019) [1,2,3]. This reported coverage was comparable to the EU average but concealed large inequalities, e.g., screening was reported only by 53% of less-educated women, in contrast to 80% for the more educated [1,2,3]. Greece met these inequalities with its first population-based program—the “Fofi Gennimata” scheme—introduced in 2022 and covering free mammography for all women between 50 and 69. This program (the nation’s first systematized cancer screening program) is attempting to improve participation levels by mailing out invitation letters and raising public awareness. Early results are encouraging (more than 300,000 mammograms were administered in the program’s inaugural year), but notifying the rest of the unscreened population remains a problem [1,4,5]. Specifically, officials have stated that ongoing communications are “one-size-fits-all” in nature and do not reach large numbers of socioeconomically and culturally diverse women. No targeted outreach is performed to high-risk subpopulations (e.g., by educational attainment, rural residents, or minorities). This communication bottleneck is the largest restriction; even if screening services are rendered at no cost, the program’s success depends on good communication that will get women in [6,7,8].

The crafting of persuasive screen messages is no less important than the clinical service itself, because persuasion cannot happen until attention to the visual is held captive first—especially in women 40+ with information overload and sometimes with cancer fear or fatalism. Unless essential content (e.g., the call-to-action to achieve a mammogram or concise declarative statements of screening advantages) is seen, its persuasiveness is lost [6,7,8]. This is particularly true for older adults, who can have an age-related decline in vision or attentional ability. Greek policymakers have therefore started to add less complex channels and formats (e.g., plain-language websites and text messages), but it is unclear what optimal combination of graphics, text, and organization would be effective [9,10]. Human–computer interaction and health communication research indicates that competing design elements differentially capture attention and structure perception—information that can be leveraged to inform message development [11,12]. Among that evidence, one repeated question is how to balance appeal on an affective plane with cues for efficacy/credibility. Fear cues are readily capable of gaining attention and, in limited-capacity and EPPM frameworks, to provoke protective response when coupled with unambiguous cues of efficacy; without that coupling; however, fear poses threats of anxiety, defensive avoidance, and message rejection—threats maximized among older adults, to whom facilitating, solution-based framing (e.g., survivor stories) is optimal [1,4]. The new consensus, then, is to produce messages that produce a sense of urgency by conveying the gravity of breast cancer and concurrently building trust in screening and in the health system—a balance of attention and motivation commensurate with early capture and follow-up steps [9,11,12].

A constant design problem is how to balance the presentation of narrative and pedagogical information in screen displays. Testimonies from people (e.g., survivor experiences) will tend to foster identification and affective involvement, and statistics and factual information enhance perceived credibility and meet analytically minded readers’ needs; in reality, the best materials exploit both using a mix of each, allowing stories to motivate and numbers to reassure [3,13]. Credibility cues—logos and approvals from credible organizations—can enhance trust, but only if noticed, and that hinges on their salience and position near the call-to-action. While that is happening, visual form needs to be age-appropriate; women ≥40 have many with declining visual acuity or lower e-health literacy, so larger type, high contrast, clear hierarchy, and brief copy help keep attention and reduce cognitive load. Nevertheless, despite large-scale cancer communication campaigns, it is rare to find PSA-specific eye-tracking research on breast screening promotion—especially in Greek/EU settings [7,8].

Most previous research compares tobacco or vaccination reminders, conflates “attention” without delineating TTFF, fixation number, and dwell, and hardly ever examines attention at AOI granularity in mixed-effects models accounting for participant–stimulus structure [3,6,8]. Design levers like image-text congruence and co-locating credibility and access information are less commonly tracked via gaze data, and layout realization (size, position, proximity) for lower-literacy or older viewers is understudied. Our research fills these lacunae by performing an AOI-level, within-subjects eye-tracking study of six breast screening PSAs aimed at Greek women ≥ 40 years, quantifying TTFF, number of fixations, and fixation duration on semantically bounded features (imagery, slogans, statistics/body text, symbols, logos, website/CTA, source/authority) and modeling influences via generalized linear mixed models with participant random effects and adjustment for AOI area and eccentricity. Empirically, we find a scan-then-read pattern whereby small, strategically placed orienting cues are fixated early, with fluent narrative/efficacy copy carrying processing forward—explaining when and where features do their work. Practically, these findings are translated into actionable recommendations for invitations, SMS landing pages, and social media advertisements in Greek and EU programs, present credibility alongside the CTA in early-attention spaces, and combine it with concise, readable efficacy copy that encourages quick understanding and follow-through [5,9,11].

Against this backdrop, the current research examines the contribution of visual design components of breast cancer screening messages to their ability to capture women’s attention and thereby the argumentative effect of messages. This study is exploratory and relies on a purposive set of six static PSAs adapted from prior campaigns. We target 40-year-old and older Greek women—the screening-eligible population destined to gain from more impactful communication. With an eye-tracking within-subjects design experiment, we showed subjects various PSA-type screening message designs and recorded their visual attention toward pre-defined message components. In particular, we pre-defined the most critical Areas of Interest (AOIs) on each message—e.g., the focal image, the tagline or slogan, the body text (statistical detail), and the logo or credit of the message. For every AOI, we recorded standard eye-tracking metrics, which are all markers of visual attention, Time to First Fixation (TTFF) on the AOI (how quickly the viewer’s attention falls on that area), Fixation Count (number of fixations on that AOI, demonstrating how frequently it caught attention), and Total Fixation Duration (summed time attending to that AOI, disclosing depth of processing). These task-based measures give us high-resolution evidence about what attracts people in and for how long, enabling us to compare, for instance, whether an emotionally engaging image is drawn in faster than a neutral one, or whether the presence of a statistical graph boosts dwell time on the text. In addition, in the analysis we use generalized linear mixed models (GLMMs) to treat the repeated-measures data—a practice suggested to overcome the inherent variability of eye-tracking experiments and enhance statistical inference. Through examining attention patterns between message designs, this study addresses the proposed need for evidence-based communication solutions to breast cancer screening in Greece.

In brief, our eye-tracking results posted modest variation at the overall content-category level but great heterogeneity at the level of individual message elements. Small-scale cues—e.g., source marks, site/CTA boxes, and brief labels—were generally viewed earlier, whereas short narrative and efficacy copy maintained attention upon arrival; logos and abstract images rarely maintained attention unless colocated with actionable information. Demographics (age, education, household) were not significantly related to gaze behavior, suggesting layout and element realization overshadowed audience segmentation in driving attention. Overall, these patterns reinforce scan-then-read and suggest the design principle of this research: engineer early visibility of plausible, actionable cues and position fluent, efficacy-rich copy near the call-to-action to translate attention into worthwhile next actions.

The rest of the paper is structured as below. Section 2 overviews the literature, beginning with theoretical premises of visual attention in health communication and then summarizing eye-tracking evidence on public service announcements. Section 3 provides the methodology, specifying its research design and apparatus, participants and sampling, stimuli and AOI schema, the eye-tracking procedure and metrics, and the statistical analysis plan. Section 4 reports empirical results—descriptive statistics, correlations, comparison by subgroups, and mixed-effects models on both AOI and AOI-category levels. Section 5 interprets the findings for existing theories and evidence. Section 6 summarizes practical implications for policymakers, health providers, business administrators, and educators. Finally, Section 7 summarizes the conclusion and its limitations with directions for future investigations.

## 2. Literature Review

### 2.1. Visual Attention in Health Communication: Theoretical Foundations

Throughout preventive health communication, attention to visual elements is the window by which messages achieve cognitive access and, eventually, persuasiveness. Eye-tracking offers immediate, time-resolved measures of said window—most commonly operationalized as how rapidly viewers look toward a location (time to first fixation), how frequently they revisit (fixation count/revisits), and how much time they spend there (total fixation duration), and thereby connects abstract design decisions to subsequent processing and behavior. An expanding literature establishes that attention is not an undifferentiated resource but is systematically determined by message characteristics (humor or fear appeals, narrative style, credibility signals), audience engagement, and visual-text congruity [14,15,16].

Affect and arousal evidence underpin two complementary pathways to attention. Humor is able to bypass defensive avoidance and enable visual inspection; in tobacco and alcohol prevention campaigns, in [17] the authors demonstrate that humor-induced comedic insights produced greater scanning, fixations, and revisits than humorous controls, and that humor diminished smokers’ avoidance of tobacco messages—a vital counter to the widely proven boomerang hazards of threat appeals among resistant populations. Conversely, fear imagery conditionally enhances intention and attention; Avery et al. [18] establish that fear images created a stronger attention-vaccination intent association, but with non-fear images, higher attention was paradoxically linked with poorer recall, showing that attention in itself is not a great proxy for persuasion or memory and may perhaps be reflective of non-productive rumination under low-threat conditions. Together, these tests are consistent with the Extended Parallel Process Model: contentious material will capture attention at first, but its persuasiveness is dependent upon efficacy information being accessible and processed, or else avoidance or superficiality will result.

A second pathway focuses on persuasion routes and processing ease. Drawing on the Elaboration Likelihood Model, Xiu et al. [19] use ERP measures to demonstrate that peripheral affective information can boost early attentional engagement (bigger N1), while central, argument-based information is more involved in late cognitive effort (bigger P3), accounting for a temporal division of labor between routes. Convergent behavioral evidence by Bullock et al. [20] shows that storytelling is more interesting than exposition, partly because it is processed more smoothly; more fluency is a mechanistic link between attention and attitude change. In online contexts, Lam et al. [21] discovered that health infographics provoke more elaboration than text messages and that ease of comprehension predicts elaboration together across formats, demonstrating that visual design is not a “peripheral” add-on but also a predictor of central processing.

Attention is also modulated by the source of the message and the framing of the message. Hsu’s [22] factorial experiment demonstrates that negative framing and celebrity endorsement increase attention but decrease recall compared to celebrity-positive framing, for experts as endorsers, framing influences over attention and recall, and high-involvement viewers distribute more gaze over expert messages with no corresponding increases in recall. Visual focus on the endorser generates attitude towards the ad, but not recall, though warning against equating attention with learning. Label disclosure and warning lettering studies advance this understanding to micro-cues: Klein et al. [23] describe how #ad identifiers are more attention-grabbing than #sponsored in teen e-cigarette marketing, and Mays et al. [24] demonstrate that color and e-cigarette warning size change reported attention and memory, but color also changes risk judgments in a counterintuitive fashion. These findings suggest that compliance and credibility cues need to be made salient Areas of Interest (AOIs) and collocated with central claims and calls-to-action, rather than forced into secondary periphery footers.

It also influences attention, the congruence between images and text, and cognitive load. In a within-subjects eye-tracking experiment on child-safety posts, Klein et al. [25] present evidence that matched image–text posts receive considerably more visual attention than mismatched posts, and visual attention to matched posts per second is linked with higher scores on measures of knowledge, direct evidence that congruence can translate attention into learning. However, more in view is not necessarily better, in embodied conversational agent virtual health consultations, Vankit et al. [15] find that adding subtitles or secondary visualizations decreases gazing to the agent (a working alliance surrogate), and that too much visual input can suppress behavioral intentions—suggesting resource trade-offs that are consistent with limited-capacity models of mediated message processing. In older adults, risk-reducing and safety-focused designs seem to be attention-grabbing; Wang et al. [14] demonstrate that natural sportscapes perceived with prevention appeals result in greater fixation numbers and durations and greater intentions, consistent with tone (assurance vs. threat) and visual context jointly regulating attention in later-life audience members.

Apart from direct gaze dynamics, a number of studies link attention to self-efficacy and engagement, the proximal cause determinants of action. Combining social cognitive theory and ELM, Zhang et al. [16] show that both central (quality of argument) and peripheral (credibility) routes of processing lead to increased self-efficacy, which in turn mediates health information systems’ behavior intention; gender as a modulator of peripheral responsiveness suggests heterogeneity in cue usage. In online doctor-choice behavior, Qin et al. [16] show how social proof (word of mouth) and credibility signals (e.g., professional avatars, output of articles) inform decisions—proof that peripheral cues can support trust in high-stakes health judgments. In their promotion of daughter-to-mother screening, Luo et al. [26] find a three–way interaction of threat, efficacy, and virality: high threat enhances engagement but does not secure recommendation intentions; a low-threat, high-efficacy, high-virality package is optimal, and involvement mediates effects—confirming attention needs to be allocated towards efficacious, socially valued action pathways.

Some tensions and gaps emerge. Most studies are outside of cancer screening (tobacco warnings, vaccine, social media health messages), so it is uncertain whether design–attention regularities will generalize to screening-promotion tasks where reassurance and procedural clarity are so strongly emphasized. First, attention is not synonymous with intention or recall [22,27], in that what one attends to and how things are arranged (congruence, hierarchy) is as important as the degree of attention aroused. Second, older women, the target audience for breast cancer screening, continue to be under-represented in experimental eye-tracking, and where included, are reported to possibly prefer safety-framed and prevention-oriented content to threat-oriented designs [14]. Lastly, methodological heterogeneity remains: studies use self-reported attention or do not align with participants, and stimulus-crossed designs, which create design effects and restrict generalizability.

Our research fills these gaps head-on by testing screening-promotion commercials on women aged ≥ 40, measuring attention to select content features (emotional imagery, narrative slogans, statistics/text density, symbols, logos) using AOI-level eye-tracking, and estimating effects with proper mixed-effects models to account for participant- and stimulus-level variation. Here, we examine whether the anticipated balance—attention capture without overload, credibility without invisibility, narrative fluency with transparent efficacy—obeys in the material landscape of design where screening options are evoked.

### 2.2. Eye-Tracking Evidence on Public Service Ads

Studies of breast cancer public service communication show a chronic disconnect between what communications intend to achieve and what the public, in fact, takes notice of. Eye-tracking studies bridge this disconnect by connecting design elements to attention and, in certain instances, knowledge or intentions and proximate content and intervention measures explaining why certain features work or fail. Together, these strands suggest that attention is not enough; it must be choreographed so attention lands on credibly presented, actionable information in front of those audiences most in need.

Two eye-tracking studies are particularly instructive for publicly addressed health communications. Klein et al. [25] demonstrate how visual-text congruence can be a powerful force in driving attention and learning: social-media public-safety messages with visually congruent material compared with the verbal advice received considerably longer looks, and additional seconds of looking boosted knowledge scores. Their within-subjects design involves the separation of a mechanism of greatest relevance to screening promotion: alignment of that which is perceived first by the audience with that which it must learn second turns attention into understanding. Yılmaz et al. [28] employ this account to complex information displays, illustrating that healthier-literate women take a longer time on hospital report cards than less literate women, and older patients have longer per-fixation dwell time with information they find critical. While the stimulus proper is not a PSA, the implication for mass campaigns is obvious: typography, hierarchy, and information chunking need to be aligned with literacy differences if attention is to center on efficacy and access cues instead of the decorative.

Surrounding these eye-tracking findings is scholarship that explains why most breast-cancer-themed advertising struggles to convey information effectively. AbiGhannam et al. [29] content analysis discovers that cause-marketing advertisements are symbol-rich (pink ribbons, survivor photos) but thin on actionable information or donations disclosure; Harvey et al. [30] also warn that pink-ribbon cause marketing can degenerate into cue-based persuasion with minimal actionable information and consumer resistance accountability. Dobrenova et al. [31] also document systematic variations in appeal use by behavior, erotic, fear, and humor appeals are more prevalent in self-examination messages than in mammography promotion messages, the latter using hardly hybrid, humor-seasoned blends. These narrative analyses are not eye-tracking, but they take into consideration why ET work tends to find gaze riveted on affect or branding cues; campaigns have long substituted front symbols for instructions, closing doors on attention to translate into self-efficacy.

Evidence on mobilizing underserved populations points to the necessity of re-targeting form and content. In Florida, Bryant et al. [32] (based on mixed methods) identify structural and cognitive drivers of screening (physician recommendation, insurance, guideline awareness, misperceptions), and propose segmented strategies combining changes in delivery of service with education tailored to segments. In Nigeria, Nelson et al. [33] demonstrate how awareness levels can be the same across areas, but sources of awareness vary widely, demonstrating that attention is influenced by message bearers, who and where, before design factors can take effect. At a more advanced level of theory, Pasick et al. [34] deconstruct individual-cognition approaches for abstracting behavior from their sociocultural setting and advocate multilevel and anthropological approaches; this is because it can account for why the same images can yield different scanpaths and interpretations within communities.

Decision support intervention trials muddy the assumed connection between more information and more screening. López–Panisello et al. [35] demonstrate that balanced information enhances knowledge but does not directly change intentions; rather, behavioral variables like anticipated regret and decisional conflict mediate change in the direction of participation. Carles et al. [36] describe a cluster-randomized trial to test whether notice of benefits and harms facilitates more informed choice, if not necessarily uptake—a difference that reframes what attention must do in ethically attentive PSAs. Adding to this, Skandali et al. [37] combine biometric emotion measurement with interviews to show fear appeals only work if coupled with coping cues; otherwise, negative affect escalates without adaptive intention. This neuromarketing research is in line with eye-tracking’s fundamental finding: attention must be designed to land on efficacy and action material right after arousal, or it will be lost to avoidance.

Cultural and religious symbols can also reinvent the path to persuasion. In a factorial study with African American women, Lumpkins demonstrates that the Christian cross enhanced ad attitudes across levels of involvement, functioning not just as a peripheral cue but as a central meaning cue. This result contradicts the easy dual-process expectations of “peripheral” design elements and indicates that, for culturally meaningful symbols, AOIs traditionally viewed as ornamental can be central sources of message understanding, a finding in line with Klein’s congruence effects [23,25] and Yılmaz et al. [28] literacy-contingent processing.

Last, systematic reviews of promotion strategies reference invitations [38,39], reminders, and psychosocial supports as uptake drivers but also detail methodological shortcomings—randomized designs, heterogeneous outcomes, and scant integration of process measures such as gaze. From an eye-tracking point of view, this is a missed opportunity, with no AOI-level data available; one cannot know whether a given PSA failed because it failed to achieve attention, because it achieved the wrong things, or because the right things were not visually available to lower-literate viewers [40,41].

Collectively, the literature implies three public service design priorities: make visual–text congruence so that attention gained results in effective content; move efficacy and access information up to high-salience AOIs, especially among lower-literacy individuals; and make appeals harmonize with community meanings so that symbols and endorsements serve as central, not surface-level, cues. Our research advances this agenda by deploying AOI-level eye-tracking to women’s 40+ targeted breast cancer screening commercials, measuring the extent to which emotional imagery, narrative taglines, stats/text blocks, symbols, and logos compete or co-allocate for attention, and experimentally testing these effects using models that appropriately control for participant and stimulus heterogeneity. By performing so, it directly addresses the discipline’s core gaps: the lack of PSA-specific ET proof for promoting screening, assessing congruence and literacy’s impact on scanpaths insufficiency, and the necessity of closing attention capture with ethically more preferable outcomes like informed decision and effective action. Structured screening programs incorporate mailed diagnostic invite mechanisms (encompassing pre-booked or optimized booking), text message reminders, phone/primary care provider-endorsed approaches, and patient-portal notification systems that aim to increase attendance. For the abovementioned notification methods, message recipients are exposed to a visual message layer (mailer/leaflet insertions, fixed PSAs/public service announcements or landing pages), wherein information must first (i) express eligibility status and action path information (availability/strategy information regarding booking terms and time) and (ii) establish credibility/data integrity (S/authority figures behind the program). This research focuses solely on this more general visual message layer, such that through the exploration of what page features trigger early orientation (time to first fixation/TTFF) and page feature use during processing (FD/FC), results apply generally in terms of letter page and linked SMS pages/PSA page design, regardless of communication medium.

Despite the existence of strong proof that mammography saves lives when performed in time, there are many women in the target age bracket who do not pay attention to or respond to screening notices and PSAs. The first objective thus involves attracting attention in the early stages of processing that are most relevant (eligibility, credibility, access), and ensuring that attention lasts long enough in support of understanding and intentions. Eye tracking provides the information of use in communication design by distinguishing the early stages of orientation (time to first fixation, TTFF), from sustained attention (fixation durations, FD), and revisits (fixation number, FC). Building on this literature, our applied objective is to identify which PSA elements and layouts most effectively attract and hold attention to the actionable content that supports screening uptake. The following research question and hypotheses are derived from the theory and evidence literature:

**RQ1.**  *Which types of visual content in breast cancer screening advertisements (e.g., text, imagery, symbols, source cues) attract the most visual attention among women aged 40 and above?*

**RQ2.**  *Do eye-tracking metrics significantly differ across AOI categories, indicating systematic differences in how participants process textual, symbolic, and emotional content?*

**RQ3.**  *How does participant age, education level, or household composition influence gaze behavior (Fixation Count, Duration, TTFF) when viewing health-related advertisements?*

**RQ4.**  *Are there significant interactions between AOI category and participant demographics in predicting gaze behavior?*

**RQ5.**  *To what extent do emotionally salient elements (e.g., human faces, self-exam gestures) outperform source or institutional logos in capturing visual attention?*

## 3. Research Methodology

### 3.1. Research Design and Apparatus

We employed a lab-based eye-tracking study to measure visual attention and emotional response to six static public service announcements (PSAs) encouraging breast cancer screening. The study was a within-subjects, repeated-measures design where all six stimuli were presented to every participant [42,43]. For reduction in order effects, advertisement presentation was randomized at the participant (balanced Latin-square where possible; otherwise, random re-ordering), a method proven to diminish sequence bias in advertising and persuasion [42,43]. Stimuli were shown full-screen for 10 s per stimulus with the stimulation interval of 5 s neutral gray sandwiched between them to constrain carryover and serve as a visual reset, simulating passive media viewing [44,45]. The sessions took place within the Laboratory of Integrated Marketing Communications, Department of Business Administration, National and Kapodistrian University of Athens, under ambient lighting control and low-noise conditions. The gaze data were captured via a Tobii Pro Nano screen-mounted eye tracker (sample rate 60 Hz), providing non-invasive, chinrest-free recording with natural head movement—suited for an ecologically valid view of the advertisements. The stimuli were projected in full-screen (borderless) mode on a 24-inch LCD display monitor (aspect ratio 16:9; 52.7 × 29.6 cm active image area) in 1920 × 1080 pixel resolution and 60 Hz refresh rate, while display scaling remained fixed at 100%. At the average viewing distance of 60 cm from the display screen, 1° of visual angle equates to 1.05 cm, and the pixel pitch of 0.277 mm approximates 0.026°/pixel. The background color throughout the inter-stimulus intervals remained mid-gray (RGB 128-128-128), and exposure durations remained fixed at 10 s per image, with or without preceding or following inter-image intervals of 5 s in neutral gray.

**Figure 1:**
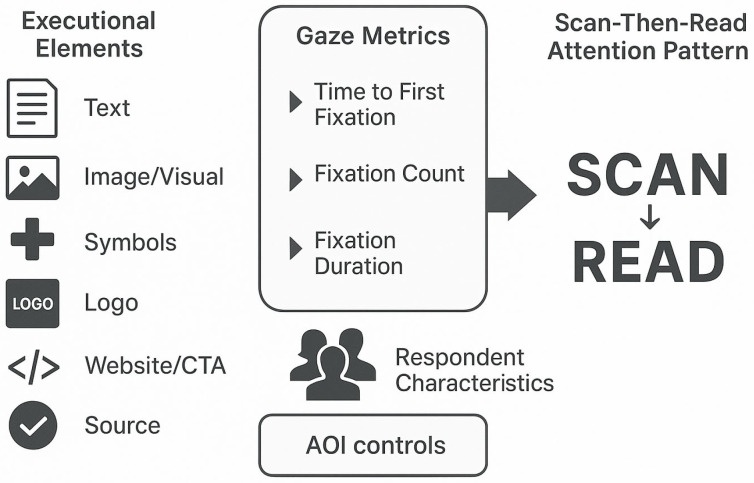
Conceptual model.

A standard 9-point calibration and validation were conducted at the beginning of each session, and participants were recorded from ~60 cm viewing distance. Facial Expression Analysis (FEA) was simultaneously recorded using iMotions v.11 to provide an index of overt affect during exposure, but was not analyzed here, as they form the basis of a companion manuscript on affect–attention coupling. This setup of equipment is optimal practice in neuromarketing and visual attention studies through combining objective measures of attention with concurrent affective measures [43,44]. Pre-defined and manually constructed Areas of Interest (AOIs) were used on each PSA to facilitate component-level analysis. AOIs were mapped to six interpretable classes to the research questions: Image/People, Slogan/Headline, Body Text/Statistics, Symbols/Object (e.g., ribbons, icons), Logo/Source, and Website/Call-to-Action. Key eye-tracking measures were Time to First Fixation (TTFF), Fixation Count (FC), and Total Fixation Duration (FD) calculated per AOI and stimulus. Figure 1 illustrates the conceptual model of this research.

### 3.2. Participants and Sampling Strategy

We employed quota-stratified purposive sampling to enroll women ≥ 40 years, the intended audience of breast cancer screening communication [46,47]. Thirty participants were enrolled from university mailing lists, local community health clinics, social media, and word of mouth. This sample is applicable to within-subjects eye-tracking and neuromarketing experiments, where 20–40 subjects can yield good power and stable estimates with high levels of repeated AOI-level observations [48].

A pre-exposure questionnaire briefly measured demographics and stratified the participants into strata. These attributes were later employed in covariate control and exploratory subgroup comparisons (e.g., multi-group analyses) when investigating gaze measures and ad ratings. Sessions took place in the Integrated Marketing Communications Laboratory of the National and Kapodistrian University of Athens. On arrival, participants were given a standardized introduction, given written informed consent in accordance with GDPR and institutional ethics protocols, and underwent a 9-point calibration on the Tobii Pro Nano (60 Hz) for accurate gaze mapping. The task was a within-subjects, repeated-measures design; all six static breast cancer screening adverts were presented full-screen to each participant for 10 s each, with a 5 s neutral gray inter-stimulus interval between them to reduce carryover and visual fatigue [49]. The order of presentation was randomized for each participant (counterbalanced where possible) to reduce sequence and order effects [42,43].

In total, 30 participants were thus used as the final sample (Table 1). To promote heterogeneity, quotas were imposed on three dimensions of demography. First, age was evenly distributed across four groups, highest being in the 40–45 and 56–60 groups (30.0% each), 51–55 (23.3%), and 46–50 (16.7%). Secondly, the majority of the subjects were married with children (63.3%), 16.7% singles without children, and 10.0% singles with children and married without children. Thirdly, in terms of education, 43.3% said they had completed compulsory high school, 23.3% said they had a university degree, 20.0% had a master’s or higher, and 13.3% said primary school was the highest level of education.

During exposure, Time to First Fixation (TTFF), Fixation Count (FC), and Total Fixation Duration (FD) were recorded for hand-coded Areas of Interest (AOIs) on all ads. AOIs were coded according to Text, Image/Visual, Symbol, Logo, Website, and Source/Authority, and attention allocation is therefore structurally comparable between content types. After eye-tracking, participants also completed a post-exposure survey measuring emotional appeal, credibility, persuasiveness, and motivational influence for each ad. The combination of objective gaze measures with subjective ratings is in accordance with best practice in neuromarketing and health communication and yields convergent evidence of implicit attention and explicit response [42,43].

### 3.3. Stimuli Materials and AOI Definition

Experimental stimuli were six professionally made, static public service announcements (PSAs) for breast cancer screening. Three were copies of successful public-health campaigns, and three were made ad hoc by creative agency DIMANA in collaboration with the research team, based on prior literature about breast cancer communication and marketing. Selection criteria were defined a priori in order to include both analysis coverage and ecological validity. Materials were selected if they contained the following: (i) relevance to screening in the presence of invitation, efficacy, or access information; (ii) message forms in terms of narrative and didactic/statistical message style; (iii) visual realization in terms of person imagery or illustration versus iconography with text; (iv) credibility information in terms of logos or sources/authorities; or (v) legibility from the perspective of minimum body text x-height of 14 px in 1080 p resolution. Materials were not selected if they contained clutter beyond analysis requirements or copyrighted brand information not applicable to screening. The final set was designed in keeping with common patterns observed in Greek/EU screening campaigns, such as survivor stories, family framing, statistical infographic cards, prevention slogans, diagnostic symbols or magnifiers, and empowerment through CTA. This serves the purpose of ensuring that the taxonomy in the AOI (Text/Information, Image/Visual, Symbol, Logo, Web/CTA, Source/Authority) corresponds with legitimate material observed in the real-world invites and PSAs that are more likely to validate the screen-eligible females in Greece (≥40).

In accordance with Section 3.1, all of the PSAs were shown full-screen for 10 s followed by a 5 s neutral gray screen to allow for a visual reset and reduce carryover effects—an exposure procedure in line with eye-tracking best practice where equal view time is needed to manage cognitive load and facilitate valid comparisons between stimuli [43,44]. All images were standardized to 1920 × 1080 px, sRGB, and normalized for global luminance/contrast within a ±5% tolerance to minimize low-level confounds (while preserving design intent). Before data collection, the contents of the six candidates were rated independently by two health communication researchers and one creative director regarding their content validity (relevance of screening cues), appropriateness of themes and material, and legibility when placed 60 cm distant from the viewer. A small pilot test (n = 6 females aged 40–60 years) verified the recognizability of central message features (logos, call-to-action, source information) and readability of headlines and text; only minute corrections in micro-contrast and text positioning were necessary.

To facilitate component-level analysis, we established Areas of Interest (AOIs) on every PSA. AOIs were hand-drawn using the Tobii Studio software v.11 interface based on human-annotation guidelines by [50]. Identical AOI boundaries were utilized across all subjects for a specific stimulus. We exported centroid coordinates and pixel area per each AOI and calculated eccentricity (screen center distance) so that these layout characteristics could be used as covariates in the modeling. Eye-tracking measures—Time to First Fixation (TTFF), Fixation Count (FC), and Total Fixation Duration (FD)—were calculated per stimulus and per AOI. In total, 41 AOIs were defined across the six PSAs and classified into six semantic categories to support structured comparisons aligned with our research questions.

**Figure 2:**
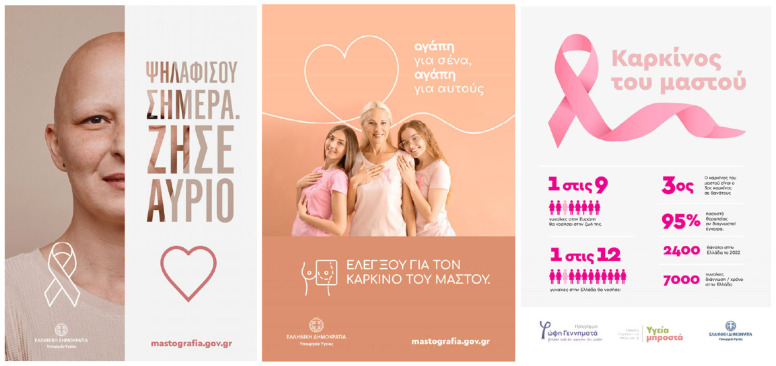
Breast cancer awareness posters shown to the participants, from left to right: Poster themed with survivorship and emphasizing optimism after treatment (**left**), Poster bearing a message of routine screening and self-examination (**middle**), Infographic-style panel showing prevalence statistics and early-detection facts (**right**).

**Figure 3:**
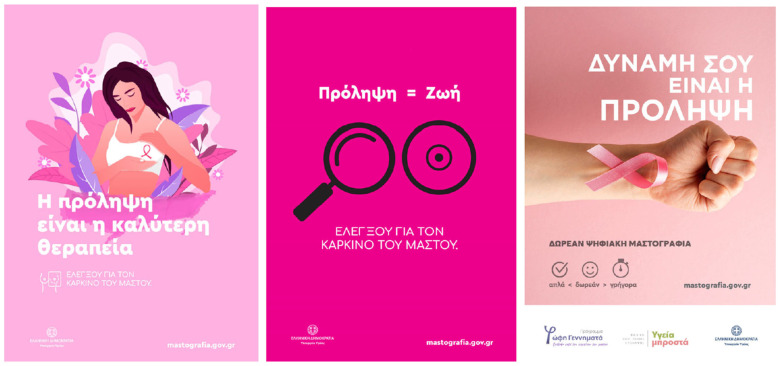
Breast cancer awareness posters shown to the participants, from left to right: Artwork emphasizing prevention as the best treatment (**left**), Graphic design using a magnifying glass to signify screening and early diagnosis (**middle**), Empowerment-themed poster promoting prevention and use of free mammography programs (**right**).

Table 2 presents a consolidation of the six PSAs for breast cancer screening that were used as stimuli, with each ad’s concept, headline, most noticeable images, and credibility/sponsor cues, as well as exemplar areas of interest. Overall, the set spans complementary design strategies—survivor narrative, family framing, fact-based messaging, self-exam iconography, strong color/objectification test, and empowerment/access information—while manipulating attention-relevant factors systematically (imagery vs. text, symbols, logos, website/CTA, and source/authority). This variation was for purposes of AOI-level comparisons of attention (TTFF, fixation frequency, fixation duration) and to determine if emotional, textual, symbolic, or institutional cues differentially capture and maintain attention in 40+-year-old women, as illustrated in Figure 2 and Figure 3.

**Figure A1:**
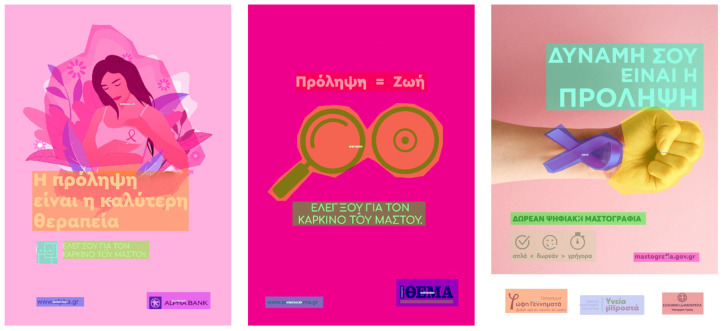
Breast cancer awareness posters with annotated AOIs. From left to right: (**left**) Survivorship/optimism poster—AOIs: HeadText (slogan), Symbol (ribbon/heart), Website/CTA, Logo. (**center**) Routine screening and self-examination—AOIs: HeadText, Image/Visual (portrait), Symbols, Source/Authority. (**right**) Infographic with prevalence and early-detection facts—AOIs: HeadText, SmallText blocks, Symbols (icons), Website, Logo/Source.

**Figure A2:**
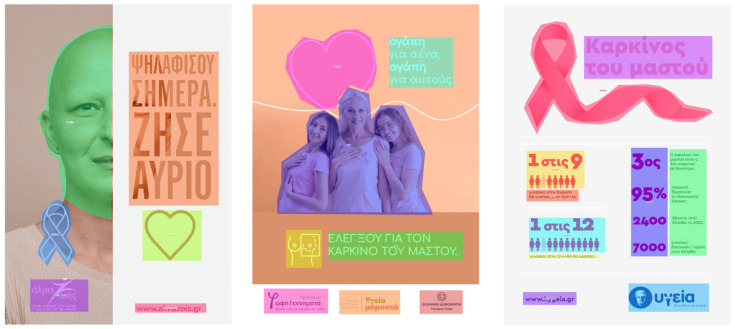
Breast cancer awareness posters with annotated AOIs. From left to right: (**left**) “Prevention is the best therapy”—AOIs: HeadText, Image/Visual (illustration), Website/CTA, Logo. (**center**) Magnifying-glass design signaling screening/early diagnosis—AOIs: HeadText, Image/Visual (magnifier), Website, Logo. (**right**) Empowerment/free mammography programs—AOIs: HeadText, Symbol (ribbon), Text-with-icons, Website/CTA, Source/Authority.

This typology permits between-content-type comparative gaze analysis and permits inferences to be made about which part of the ad was most focused upon. All AOIs were labeled onto a standardized template (e.g., Ad3_SmallText2) so that it could be combined with participants’ fixation streams of data and cross-tabulated with demographic measures and post-exposure rating scores. By categorizing AOIs in this way, the research enables both descriptive identification (e.g., which aspects drew gaze reliably) as well as inferential hypothesis testing (e.g., AOI-level measurements of gaze and effects on persuasion correlation). Such systematic classification is in line with best practice within neuromarketing research and improves the reproducibility and interpretability of eye-tracking outcomes. A summary of all AOIs defined, and ad assignments and semantic categories, is given in Appendix A, Table A1 (Figure A1 and Figure A2). The Table will serve as a reference for describing gaze measures in the following paragraphs.

### 3.4. Eye-Tracking Procedure and Metrics

This paper focuses only on the inferential testing of eye tracking variables (Time to First Fixation, Fixation Count, and Fixation Duration) conducted through the Area of Interest (AOI) level. The only use of pre-exposure demographics in this study is through covariate analysis in subgroup testing; post-exposure rating data and facial expression (FAC) data streams are obtained only for quality control and preregistered archival procedures and are not analyzed in this paper, but are relevant to another manuscript regarding affect-attention couplings. All the experiments were administered singly in the Integrated Marketing Communications Lab using ambient-controlled lighting, wall color, and low background noise to minimize extraneous load. The participants reclined at ~60 cm distance from the screen, with free head movement maintained, while tracking accuracy was ensured. A Tobii Pro Nano eye tracker (60 Hz) was placed below the screen and was connected to iMotions for presenting the stimulus, calibration, and data collection. Prior to exposure, all participants completed a standard 9-point validation calibration. Data-quality targets were accuracy ≤ 1.0° and precision (RMS) ≤ 0.5°; recalibration was performed if thresholds were not met. Valid sample ratio per trial had to be ≥70%. Spatial precision for AOI-based analyses was ensured through continuous tracking and recalculation when the average error exceeded 0.5° visual angle. Participants then watched six static breast cancer screening adverts full-screen for 10 s each, with an intervening 5 s neutral gray screen to facilitate visual reorientation and reduce carryover (see Section 3.1). Gaze was monitored continuously during.

We quantified attention at the AOI level using the following three standard metrics:

- Time to First Fixation (TTFF) (ms): latency from stimulus onset to the first fixation within the AOI; an index of initial salience/attentional pull.
- Fixation Count (FC) (n): number of fixations within the AOI; a proxy for revisitation and processing episodes.
- Total Fixation Duration (FD) (ms): cumulative fixation time within the AOI; an indicator of depth of processing and/or affective engagement.

Fixations were identified using Tobii’s I-VT algorithm (velocity-threshold identification; 30°/s threshold), with a minimum fixation duration of 60–80 ms and standard merging criteria for adjacent fixations within 0.5°/≤75 ms [50]. Short gaps (<75 ms) were interpolated; blinks and samples flagged as invalid were removed. AOIs were predefined as non-overlapping polygons; the fixation centroid rule was used to assign each fixation to at most one AOI. Fixations were detected employing the I-VT algorithm developed by Tobii (Velocity-Threshold Identification; 30°/s threshold), requiring a minimum fixation duration of 60–80 ms and standard merging criteria regarding adjacent fixations no larger than 0.5°/≤75 ms. Gaps under 75 ms were interpolated; blink and invalid samples were erased. The regions of interest consisted of predefined polygons that did not overlap. The fixation point defined each fixation’s association with one region of interest. TTFF was calculated as the time from the onset of the stimuli to the onset of the first fixation whose centroid fell inside the region of interest; regions of interest that were not fixated in 10 s were censored at 10,000 ms. FC represented the number of discrete fixation events for the AOI based upon the above-mentioned rules of merging; re-entries (leaving and later re-fixating the AOI) increased the count. The FD value represented the sum of the durations (in milliseconds) of all fixations in the AOI; saccades and fixation samples were not included. Fixations across boundaries received assignments based upon location (no splits in time). Cases were dropped if valid samples were < 70%, calibration drift > 1°, or if attention to the screen was not attended. Exporting data was in long form (participant × ad × AOI). The current operationalization aligns with often-quoted recommendations on fixation-related metrics and detection in static-image trials and with the technical specification I-VT in the manufacturer’s I-VT [50]. The interpretation of the metrics TTFF, FC, and FD regarding the usage of multiple dimensions of orienting and sustained processing corresponds with common outcomes of classic eye tracking research in vision and in marketing-related areas [46,47,50,51]. This methodology is optimal practice in health-message research and neuromarketing by virtue of bringing together objective, non-verbal measures of attention with tightly controlled viewing conditions [46,47,50,51].

## 4. Data Analysis and Results

### 4.1. Statistical Analysis Plan

Analyses were conducted in three phases described a priori in Google Colab [52]. In the first, we created descriptive summaries of participant demographics (age, education, household structure) and estimated AOI-level means/medians of time to first fixation (TTFF), fixation count (FC), and total fixation duration (FD) by AOI category (Text, Image/Visual, Symbol, Logo, Website/CTA, Source/Authority) in order to draw an initial map of attention deployment between content types. Second, to investigate demographic differences without parametric statistic assumptions, we pooled AOI metrics to the participant level and conducted Kruskal–Wallis tests across age groups, education levels, and household groups; significant omnibus tests (α = 0.05, two-tailed) were followed up using Dunn’s post hoc contrasts with Bonferroni/Holm corrections [53,54]. Third, main inferences were based on generalized linear mixed models (GLMMs) with crossed random effects of participants and ads to control for the repeated-measures design [55,56,57,58]. All variables were modeled with a distribution suitable to their scale and skew, FC as count response using the negative binomial family and log link (to handle overdispersion), FD as gamma with log link or log-normal (chosen through AIC/BIC and residual plots), and TTFF as log-normal for fixated AOIs, accelerated failure-time (AFT, log-normal) survival model applied as robustness check to handle right-censored AOIs not assigned any fixation during the 10-s exposure [59,60,61]. Fixed effects were AOI category and demographics (household composition, age, education), with AOI category by demographic interactions to account for RQ4.

Where possible, by-participant random slopes of AOI category accounted for participant-specific scanning. AOI area (pixels or proportional area) and eccentricity (distance from screen center) were controlled in order to account for size/position effects, where possible; face-present and text-density (characters/cm^2^) were examined as ancillary covariates. Omnibus effects were evaluated with likelihood-ratio tests, and pairwise contrasts employed estimated marginal means (EMMs) with Holm adjustment. The effect sizes are given as incidence-rate ratios (IRR) for FC, and geometric mean ratios for FD and TTFF, with 95% confidence intervals, marginal/conditional R^2^, intraclass correlations (ICC), and overdispersion diagnostics. According to RQ5, planned contrasts between Image/Visual and Logo, Slogan/Headline and Logo in FC and FD, and quicker orienting on Image/Visual or Slogan/Headline compared to Logo in TTFF; theory-matching contrasts also between Symbol and Image (TTFF longer; FC/FD lower). Model fit was verified by residual plots, alternative link/family sensitivity, and exclusion of low-quality trials.

Our design provides a large number of within-participant and across-stimuli in the AOI level (30 participants × 6 ads × 41 AOIs), contributing significantly to the information available on fixed effects even in relatively small samples. Mixed-effects models leverage this data structure by decomposing the deviance into levels of participants and items/ stimuli, improving precision in fixed-effect estimation and maintaining type I error rates in crossed-random-effect vision/psychophysical research. Theoretical analysis and computer simulations reveal that linear mixed models or generalized linear mixed models are able to provide sufficient power with 20–40 participants when considering (a) Within-subject research questions, (b) Many trials or stimuli per participant, and (c) random intercepts/slopes capture clustering appropriately. We thus model AOI-level metrics with GLMMs and robust SEs, prioritizing within-subject contrasts and reporting EMMs with 95% CIs. Specifically, the model specifications (base GLMMs):

Let $y_{p,a,c}^{\mathrm{FC}}$, $y_{p,a,c}^{\mathrm{FD}}$ and $y_{p,a,c}^{\mathrm{TTFF}}$, denote, respectively, fixation count, total fixation duration, and time to first fixation for participant *p*, ads *a*, and AOI category *c*. Let ${Area}_{p,a,c}$ be AOI pixel area, ${Ecc}_{p,a,c}$ its eccentricity, and $X_{p}$ the demographics covariates (age, education, and household). In particular:

1. **Fixation Count (negative binomial, log link):**

$$\log E\left[y_{p,a,c}^{\mathrm{FC}}\right]=\;\beta_{0}+\;\beta_{c}\;\mathrm{AOIca}t_{c}+\;\beta_{X}^{⊤}X_{p}+\;\beta_{c\;\times \;X}^{⊤}⊤\left(\mathrm{AOIca}t_{c}\;\times \;X_{p}\right)+\;\log\left(\mathrm{Are}a_{p,a,c}\right)+\;\beta_{E}\;\mathrm{Ec}c_{p,a,c}+\;u_{p}+\;u_{a},$$

with $u_{p}∼N\left(0,\;\sigma_{u}^{2}\right),\;v_{a}\;∼\;N\left(0,\;\;\sigma_{v}^{2}\right)$, where $\;\beta_{0}$: Intercept, $\beta_{c}\;\mathrm{AOIca}t_{c}$: Category effect, $\beta_{X}^{⊤}X_{p}$: Vector of participant-level predictors, $\beta_{c\;\times \;X}^{⊤}⊤\left(\mathrm{AOIca}t_{c}\;\times \;X_{p}\right)$: Interaction between AOI category and predictors, $\log\left(\mathrm{Are}a_{p,a,c}\right):$ Offset term, $\beta_{E}\;\mathrm{Ec}c_{p,a,c}$: Effect of eccentricity, $u_{p},u_{a}$: Random effects for participant p and age group a.

2. **Fixation Duration (gamma with log link or log-normal):**

$$gE(\left[y_{p,a,c}^{\mathrm{FD}}\right])=\;\beta_{0}+\;\beta_{c}\;\mathrm{AOIca}t_{c}+\;\beta_{X}^{⊤}X_{p}+\;\beta_{c\;\times \;X}^{⊤}⊤\left(\mathrm{AOIca}t_{c}\;\times \;X_{p}\right)+\;\beta_{A}\left(\mathrm{Are}a_{p,a,c}\right)+\;\beta_{E}\;\mathrm{Ec}c_{p,a,c}+\;u_{p}+\;u_{a},$$

where $g\left(\cdot \right)=\log\left(\cdot \right)$ for gamma, for log-normal specification, log $\left(y_{p,a,c}^{\mathrm{FD}}\right)$ is modelled with an identity link and normal residuals.

3. **Time to First Fixation (log-normal GLMM for fixated AOIs):**

$$log\left(y_{p,a,c}^{\mathrm{FC}}\right)=\;\beta_{0}+\;\beta_{c}\;\mathrm{AOIca}t_{c}+\;\beta_{X}^{⊤}X_{p}+\;\beta_{c\;\times \;X}^{⊤}⊤\left(\mathrm{AOIca}t_{c}\;\times \;X_{p}\right)+\;\beta_{A}\left(\mathrm{Are}a_{p,a,c}\right)+\;\beta_{E}\;\mathrm{Ec}c_{p,a,c}+\;u_{p}+\;u_{a}+\varepsilon_{p,a,c},$$

where $\varepsilon_{p,a,c}∼N\left(0,\;\sigma^{2}\right)$. As a robustness check, TTFF was also analyzed via a log-normal AFT survival model including right-censored AOIs (no fixation within 10 s).

### 4.2. Descriptive Statistics

We had N = 1230 AOI-level measures to operationalize primary eye-tracking measures and subject attributes. Mean TTFF over all AOIs was 641.29 ms (SD = 1644.72), and mean total FD was 289.16 ms (SD = 449.97). Both TTFF and FD were highly non-normal (Shapiro–Wilk *p* < 0.001), with highly positive skew and leptokurtosis (TTFF skew = 3.56, kurtosis = 13.01; FD skew = 12.20, kurtosis = 199.95), indicating frequent short-latency/short-dwell and infrequent long latency and long dwells. As would be expected in a stationary 10-s fixation, maxima neared the edge of view (TTFF ≈ 10,000 ms; FD ≈ 9483 ms), as would be expected under right-censoring for AOIs not fixated until towards the end of the interval.

The Fixation Count (FC) distribution was also non-normal (Shapiro–Wilk *p* < 0.001) and showed moderate overdispersion for a per-AOI event count (M = 1.34; SD = 1.28; median = 1; IQR = 2). These values reflect discrete fixation events per AOI per trial (I-VT; 60–80 ms minimum fixation), not raw gaze samples.

Participant covariates were approximately evenly balanced across the pre-specified strata (household type, education, age bands). Since all three of the gaze measures did not pass normality tests (Shapiro–Wilk *p* < 0.001), and some of the grouping variables are ordinal, non-parametric tests were employed for exploratory subgroup comparisons, and distribution-appropriate GLMMs for inferential analysis (Table 3).

### 4.3. Bivariate Relationships and Correlations

Spearman’s rank correlations between the gaze measures—Fixation Count (FC), Time to First Fixation (TTFF), and Fixation Duration (FD)—and the individual characteristics (household, age group, level of education) were calculated using Spearman rank correlations (N = 1230 AOI-level observations). As can be observed, FC and TTFF were moderately and positively correlated (ρ = 0.316, 95% CI [0.264, 0.365], *p* < 0.001), which means that later AOIs in the viewing episode tended to get fixated more times. This fits expectations of such designs under which visual priority is initially accumulated toward emotive images and then following text-dense areas that—albeit with longer orienting latencies—are repeatedly examined after the viewer has switched to reading. Both FC and FD were also significantly correlated (ρ = 0.213, [0.159, 0.266], *p* < 0.001), as one would expect from the intuitive relationship between increased visit numbers and increased cumulative dwell. TTFF and FD were not significantly correlated (ρ = 0.040, [−0.016, 0.096], *p* = 0.161), indicating that arrival time at an AOI is not by itself a good predictor of processing depth.

Demographics and gaze measure correlations were consistently small and non-significant (all |ρ| ≤ 0.024, *p* ≥ 0.184), reflecting weak zero-order relations between household type, age group, or education level with composite measures of attention—a finding further pursued with covariate-adjusted mixed models in Section 4.5. Interrelation among demographic factors revealed a moderate negative interrelation between household and age (ρ = −0.366, [−0.413, −0.316], *p* < 0.001), a small positive interrelation between household and education (ρ = 0.151, [0.096, 0.205], *p* < 0.001), and a weak negative interrelation between age and education (ρ = −0.082, [−0.138, −0.027], *p* = 0.004) according to expected cohort and life-stage relations.

Since these correlations condition on AOI observations as independent and do not control for AOI size/position or stimulus/participant clustering, these are most accurately described. The reported inferential tests below include AOI area and eccentricity, and crossed random effects for participants and ads in order to formally test the hypothesized relationships here (Table 4).

### 4.4. Inferential Statistics and Group Comparisons

#### 4.4.1. Per Household

To explore differences in gaze patterns across demographic subgroups, a set of non-parametric tests was conducted. Specifically, Kruskal–Wallis tests were conducted to assess whether TTFF, Fixation Count, or Fixation Duration differed significantly by age group, education level, and household status. Where variation is found to be significant (*p* < 0.05), Dunn’s post hoc tests with Bonferroni correction were conducted to identify the source of the variation (see Appendix A, Table A2).

For Fixation Count, a Kruskal–Wallis test indicated that there was no age group difference in the number of fixations, H(3) = 1.635, *p* = 0.651, with a non-significant effect size (ε^2^ = 0.001, 95% CI [<0.001, 0.012]). Post hoc Dunn’s tests with Bonferroni correction indicated no difference between any two age groups (all adjusted *p*-values > 0.999). A Kruskal–Wallis test revealed no household type difference in the number of fixations, H(3) = 3.009, *p* = 0.556, with a trivial effect size (ε^2^ = 0.002, 95% CI [<0.001, 0.015]). None of the pairwise household group differences were statistically significant in Dunn’s post hoc tests with Bonferroni adjustment (all adjusted *p*-values > 0.999). A Kruskal–Wallis test indicated no differences in fixation count by education level. H(3) = 0.948, *p* = 0.814, with a trivial effect size (ε^2^ = 0.001, 95% CI [<0.001, 0.010]). No differences between any of the education levels were found in pairwise comparisons via Dunn’s test with Bonferroni correction.

For Time to First Fixation, Kruskal–Wallis test for Time to First Fixation (TTFF) between Household types tested was: H(3) = 1.856, *p* = 0.762, Rank ε^2^ = 0.002, 95% CI [0.0006, 0.014]. It indicates that there is no statistically significant TTFF difference among household groups. All pairwise post hoc tests by Dunn’s test were also non-significant (all adjusted *p* > 0.05). The effect sizes (r_s_) are very small and range from 0.021 to 0.095.

Kruskal–Wallis test of Time to First Fixation (TTFF) by Age was H(3) = 1.947, *p* = 0.584, Rank ε^2^ = 0.002, 95% CI [0.0002, 0.012], and indicated no difference in TTFF among the four age groups. Dunn’s Post Hoc Tests, All adjusted *p*-values (Bonferroni and Holm) were not significant (p_adj = 1.000). Effect sizes (rank-biserial correlation) were extremely small, 0.016 to 0.061.

To contrast potential variance in Time to First Fixation (TTFF) according to levels of education, a non-parametric Kruskal–Wallis H test was utilized with a view to non-normality in the distribution of TTFF scores. Education had four groups: Compulsory/High School, Primary School, University, and Master’s Degree and above. The Kruskal–Wallis test for differences in TTFF according to levels of education was not statistically significant, H(3) = 2.656, *p* = 0.448. The effect size was very small (Rank ε^2^ = 0.002, 95% CI [4.654 × 10^−4^, 0.013]) and showed that there was a practically non-significant proportion of variance in TTFF accounted for by level of education. Dunn’s pairwise comparisons with Bonferroni and Holm corrections did not reveal differences between any two education groups. All the effect sizes (rank–biserial correlations) were trivial and varied from 0.016 to 0.076 and described trivial group differences.

For Fixation Duration by Household Type, a Kruskal–Wallis H test was conducted to investigate Fixation Duration among four household types (MarriedWithKids, MarriedWithoutKids, SingleWithKids, SingleWithOutKids). The test indicated a significant difference, H(4) = 9.762, *p* = 0.045. The effect size was small with Rank ε^2^ = 0.008 (95% CI [0.003, 0.026]), suggesting that household type explains a small explanatory power for variance in duration of visual attention. Dunn’s pairwise contrasts (Holm corrected and Bonferroni) revealed one significant contrast: SingleWithKids had longer fixation times than SingleWithoutKids, z = 2.707, *p* = 0.007, rₚ_s_ = 0.289. The contrast was marginally non-significant after correction (*p*(Holm) = 0.068), and requires caution in interpretation.

#### 4.4.2. Per Age Group

We compared demographic subgroups’ fixation behavior to participant-level aggregates (median AOI value per participant) and Kruskal–Wallis tests for Time to First Fixation (TTFF), Fixation Duration (FD), and Fixation Count (FC). After the omnibus tests achieved α = 0.05, Dunn post hoc contrasts with Bonferroni/Holm corrections and rank-based effect sizes (ε^2^ for omnibus; rank-biserial rrb for pairwise) are reported (see Appendix A, Table A3).

The Kruskal–Wallis H test compared Fixation Duration in four age groups (40–45, 46–50, 51–55, and 56–60). The test approached statistical significance, H(3) = 6.809, *p* = 0.078, with a small effect size (Rank ε^2^ = 0.006, 95% CI [0.001, 0.019]). Between-group differences were not statistically significant at the 0.05 level overall, although post hoc tests indicated two contrasts between ages of potential interest.

FD differences across the four age bands (40–45, 46–50, 51–55, 56–60) were not statistically significant at the omnibus level (H(3) = 6.809, *p* = 0.078, rank ε^2^ = 0.006, 95% CI [0.001, 0.019]). Two unadjusted Dunn contrasts were suggestive but did not survive multiplicity control, 46–50 vs. 51–55 (z = −2.403, *p* = 0.016) and 51–55 vs. 56–60 (z = 1.911, *p* = 0.056). Taken together, there is no reliable age effect on FD once family-wise error is controlled, though a modest midlife shift (46–50 vs. 51–55) is a plausible trend to probe in adjusted models.

#### 4.4.3. Per Education Level

A Kruskal–Wallis H test is employed to compare differences between four levels of education in Fixation Duration (Compulsory/High School, Primary School, University, and Master’s or more) (see Appendix A, Table A4).

The result was close to significance, H(3) = 6.667, *p* = 0.083, and with an extremely small effect size (Rank ε^2^ = 0.005, 95% CI [0.001, 0.019]). Dunn’s post hoc comparisons revealed only one contrast of interest: Master’s or higher level educated subjects had significantly longer fixation times than subjects educated to the Primary School level, z = 2.522, *p* = 0.012, with rank-biserial correlation (rᵣb) = 0.140. The difference was significant after Bonferroni and Holm adjustment (*p* = 0.070), with a moderate educational effect on visual attention. All other contrasts were non-significant. These findings imply that a higher level of education can be associated with higher visual processing in breast cancer screening ad judgment, possibly as a reflection of cognitive elaboration or ad content engagement.

### 4.5. Inferential Modeling of Gaze Metrics Across Demographics (Via Generalized Linear Mixed Models)—AOI Category Comparisons

In order to examine whether patterns of visual attention between participants differed as a function of demographic variables, the research employed a series of Generalized Linear Mixed Models (GLMMs) that treated each eye-tracking metric as a unique dependent variable. Application of such models was to be preferable to that of traditional multivariate procedures (e.g., MANOVA) since data were structured as repeated-measures, had non-normal outcomes, and individual-level variation had to be controlled for [58,62,63].

Three simple visual attention measures were explored: Time to First Fixation (TTFF), Fixation Count, and Fixation Duration. Two models of GLMM were used for each of these measures. The first model used Area of Interest (AOI) as a fixed factor and demographic controls: Age Group, Household Composition, and Education Level. The second set of models replaced single AOIs with broader AOI Categories (e.g., Text, Image, Symbol, Source/Authority) to enable the comparison of higher-order visual content structures across participant subgroups. The random intercept for Participant ID was added to each of the model sets to enable repeated measures within participants [55,56].

Both models were estimated with a log link function and gamma distribution, suitable for the right-skewed nature of the gaze measures. Fixed effects were tested with likelihood-ratio tests (Type III Wald χ^2^), and post hoc pairwise contrasts were conducted by means of estimated marginal means (EMMs) to identify which AOIs or subgroups by demographics were accountable for differences [55,56]. The GLMM model was employed since it is robust and capable of handling missing data, unbalanced group size, within-subject heterogeneity, and violations of the independence assumption. The technique supports estimation of valid participant and ad content effects on attentional outcomes as well as clustering adjustment of eye-tracking data. Inferential model findings—i.e., main effects and subgroup differences—are later discussed in the following Results section and describe how different features of breast cancer awareness ads were visually processed in subgroups of older female participants.

#### 4.5.1. Fixation Count × AOI Category

We have estimated Fixation Count (FC) at the AOI level by using a generalized linear mixed model (GLMM) with gamma distribution and log link, with AOI category (Image/Visual, Text, Symbol, Logo, Website/CTA, Source/Authority) as fixed effect and random participant intercept for handling repeated measures. Demographic covariates (age, education, household composition) were treated as fixed effects. By-participant slopes for ad category AOI and by-ad level random effects are analyzed but removed as they resulted in singular fits with the existing sample and six-stimulus design (Table 5).

The omnibus effect of the AOI category was in the direction of significance, χ^2^(5) = 9.96, *p* = 0.076 (Table below). Demographic covariates were uncorrelated with FC (all *p* > 0.39). Under the AOI factor, Symbol AOIs had lower estimated fixations count than the grand mean (B = −0.164, SE = 0.072, t = −2.283, *p* = 0.022), but others were not significantly different at α = 0.05 (Logo: *p* = 0.065; Text: *p* = 0.628; Website/CTA: *p* = 0.124; Source/Authority: *p* = 0.278). Since the omnibus test was not significant, category-by-category inferences should be regarded as exploratory and are to be interpreted with reservation in respect of multiplicity.

Estimated marginal means (response scale) supported the following descriptive rank: Image AOIs received the highest fixations (M = 2579.21), and then Text and Website/CTA; Symbol, Logo, and Source/Authority received relatively fewer fixations, with the fewest being Source/Authority (M = 1900.14). At a substantive level, the ranking aligns with attention being initially attracted to imagery/people and blocks of readable text/CTAs, and away from institutional signs and abstract symbols.

As the AOI main effect was not family-wise significant, the Symbol contrast (*p* = 0.022) must be regarded as hypothesis-generating. Second, FC is a count outcome; a negative binomial GLMM with log link is typically preferred for overdispersed fixation counts. As a robustness check, refitting with a negative binomial family and adding AOI area (as offset or covariate) and eccentricity would render causal attribution to content type more robust to size/position. Results here nonetheless provide the identical descriptive signal—Images/Text/CTAs > Symbols/Logos/Sources—that concurs with our RQ1/RQ5 expectations.

**Figure 4:**
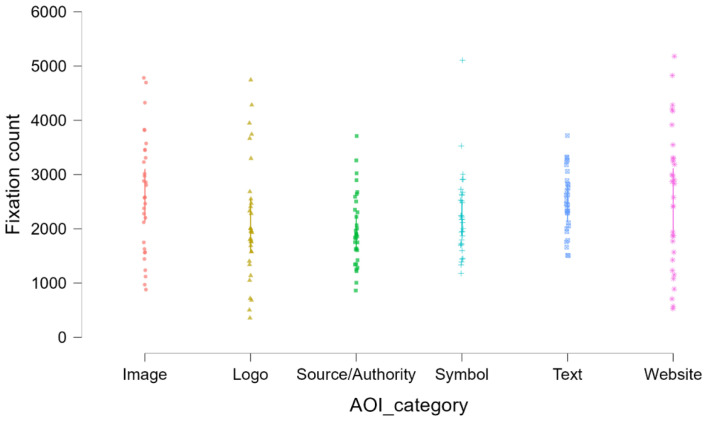
Distribution of Fixation Count Across AOI Categories.

Jittered dot plots show raw fixation count data by area of interest (AOI) category. Each observation is represented by a point colored and shaped by AOI. Image AOIs provoked the most fixations, followed by Text and Website. Source/Authority and Symbol AOIs garnered relatively fewer fixations in comparison. These trends graphically reinforce the generalized linear mixed model’s findings of a significant effect for Symbol AOIs (*p* = 0.022) (Figure 4).

#### 4.5.2. AOI-Level Fixation Count Differences

We fit AOI-level Fixation Count (FC) for the six breast cancer PSAs with a generalized linear mixed model (GLMM) with log link and gamma mean–variance relationship, with AOI (41 levels) as a fixed effect and participant as a random intercept. Household, Age, and Education were included as fixed covariates. By-participant random slopes for demographics are not estimable (no within-participant variation) and were excluded to prevent singular fits (Table 6).

At the omnibus level, AOI differed significantly in FC, χ^2^(40) = 99.79, *p* < 0.001, indicating considerable variance in the degree to which specific features within ads were revisited. No demographic covariate predicted FC (all *p* > 0.26). This pattern indicates that the layout and content of individual AOIs, more so than participant demographics, powerfully determined revisitation.

Estimated marginal means (EMMs; response scale) indicated large heterogeneity among AOIs. The most viewed component was Ad2_Icon(heart) (EMM = 4227.95, SE = 790.31; GLM estimate = +0.654, *p* < 0.001), followed by Ad1_HeadText (EMM = 3498.05, SE = 650.98; estimate = +0.464, *p* = 0.009), Ad4_Text (EMM = 3384.90; estimate = +0.406, *p* = 0.023), and Ad4_HeadText (EMM = 3302.05; estimate = +0.431, *p* = 0.016). Conversely, some of the AOIs received fewer revisits than the grand mean and these included Ad4_Icon(breast) (EMM = 1110.44; estimate = −0.613, *p* < 0.001), Ad6_FofiGenimata (EMM = 1589.28; estimate = −0.683, *p* < 0.001), Ad5_HeadText (EMM = 1276.46; estimate = −0.544, *p* = 0.002), and Ad2_Kivernisi (EMM = 1480.82; estimate = −0.396, *p* = 0.028). Substantively, what these findings show is that good narrative/affective anchors (e.g., the heart symbol in a family-themed ad and large headline/text areas) prompted more revisits, while institutional/political source caution messages and small abstract symbols underperformed. The below-mean performance of Ad5_HeadText—despite its bold color scheme—suggests color alone is not enough to support revisitation when the headline competes with other dominant images (e.g., the magnifier–breast visual). In addition, FC is a count variable; a negative binomial GLMM with log link, being in general preferable for fixation counts, ideally controlling for AOI area (offset or covariate) and eccentricity. The gamma-link estimates here therefore give us a convergent descriptive hypothesis–affective/narrative anchors and large text blocks are re-visited more than institutional/symbolic aspects-to be balanced against category-level analysis.

#### 4.5.3. Time to First Fixation × AOI Category

We modeled Time to First Fixation (TTFF) with a mixed-effects model suitable for right-skewed latencies. As part of our preregistered strategy (§3.5), TTFF was modeled in a log-normal GLMM (participant random intercept) with AOI category (Image/Visual, Text, Symbol, Logo, Website/CTA, Source/Authority) and demographic covariates (age, education, household). Robustness check (not reported), an AFT log-normal survival model employed non-fixated AOIs in the 10-s exposure as right-censored (Table 7).

The AOI category impact on TTFF was not significant (Type-III LR test: χ^2^(5) ≈ 0, *p* = 1.000), and none of the demographic covariates approached significance (all *p* ≥ 0.13). Because there is a null omnibus, we are not inferentially justified in reporting pairwise contrasts; we thus report estimated marginal means (EMMs) descriptively to summarize orientation patterns. Although the category effect for AOI in aggregate was not statistically significant, χ^2^(5) = 0.000, *p* = 1.000, estimated marginal means (EMMs) did reflect significant differences in gaze initiation patterns. The subjects first gazed most quickly at Website aspects (EMM = 291.32 ms, SE = 88.62) and Source/Authority (EMM = 343.84 ms, SE = 63.66), suggesting relatively high attentional salience in those content categories. In comparison, Symbolic elements (EMM = 688.56 ms, SE = 120.97) and Text blocks (EMM = 650.23 ms, SE = 120.29) exhibited the largest and earliest fixations and generated considerably larger TTFF estimates than the grand mean (Estimate = 0.442, *p* = 0.006 and Estimate = 0.385, *p* = 0.024, respectively). These results indicate that emotional or symbolic stimuli can maintain attention after focus has been established, but they do not necessarily capture the attention of the observer in breast cancer awareness advertisements.

The rank order of description follows a scan-then-read sequence: first, observers try action/navigation and source cues, then imagery, and finally logos, text blocks, and abstractions. However, (i) the null omnibus precludes claims of consistent between-category differences; (ii) TTFF is influenced by AOI size and eccentricity and by the presence or absence of a face—both of which we control for in the GLMMs elsewhere; and (iii) short exposure (10 s) and right-censoring can result in higher TTFF variance for low-salience AOIs. For completeness, the AFT survival re-fit provided the same qualitative ranking with no apparent AOI effect in supporting the conservative finding that systematic TTFF differences by category were not found in this sample.

#### 4.5.4. AOI-Level Time to First Fixation Differences

We compared AOI-level variation in Time to First Fixation (TTFF) on the six PSAs via a generalized linear mixed model (GLMM; log link, gamma mean–variance) with AOI (41 levels) as a fixed effect of interest and participant as a random intercept, household, age, and education as covariates. The AOI effect was large, χ^2^(40) = 268.14, *p* < 0.001, in the sense that attention onset differed for certain items across the ads, and none of the demographic covariates correlated with TTFF (all *p* > 0.35) (Table 8).

Several AOIs were observed to draw participants’ fixations much faster or slower than the grand mean. The fastest initial fixations were for text and symbol AOIs such as Ad6_Headtext (EMM = 52.83 ms; Estimate = –2.714, *p* < 0.001), Ad2_FofiGenimata (EMM = 30.65 ms; Estimate = –2.546, *p* < 0.001), and Ad3_Icon(ribbon) (EMM = 25.91 ms; Estimate = –1.268, *p* < 0.001). In comparison, comparatively slower gaze onset was associated with emotionally arousing or texturally complex areas such as Ad2_Icon(heart) (EMM = 1201.38 ms; Estimate = 1.621, *p* < 0.001), Ad1_HeadText (EMM = 1268.18 ms; Estimate = 1.317, *p* < 0.001), and Ad3_Icon3Statistics (EMM = 1459.11 ms; Estimate = 1.214, *p* < 0.001). These results show that symbol or name-based stimuli evoke attention rapidly, but emotionally engaging or text-dense stimuli attract initial attention at a slower pace.

AOIs related to program/source names (e.g., Fofi Genimata), logos/ribbons, or brief headline segments were found to draw earlier fixations, while emotionally appealing images (e.g., heart symbol in a family advert) and text-heavy/statistical blocks manifested later orienting. This order is generally in line with a scan-then-read order and with competition between salient features, certain graphic devices (ribbons/logos) and brief text anchors serve as early orienters, whereas larger headlines and data blocks are accessed later but dwelled on more extensively once accessed.

#### 4.5.5. Fixation Duration × AOI Category

We modelled Fixation Duration (FD) by AOI categories with a GLMM with a gamma distribution and a log link, with AOI category (Image/Visual, Text, Symbol, Logo, Website/CTA, Source/Authority) as a fixed effect; age, education, and household as covariates; and a participant random intercept to model repeated measures. The AOI category effect was not significant, χ^2^(5) = 7.36, *p* = 0.196, and none of the demographic covariates approached significance (all *p* > 0.21) (Table below). So, although individual features do vary in how soon they draw gaze (TTFF) or how frequently they are revisited (FC), the duration spent per visit did not vary reliably by broad content category in this sample (Table 9).

The AOI category effect was not significant, χ^2^(5) = 7.36, *p* = 0.196, with no ad element categories differing in the length of attention received. Estimated marginal means were fixation lengths from 251 ms (Website) to 309 ms (Source/Authority), with Logos (M = 260.79 ms, SE = 24.71) having comparatively longer fixation lengths than the grand mean (Estimate = 0.084, *p* = 0.089). No group differences within any household, age, or education groups were stable (ps > 0.07), reflecting attention duration invariance across groups.

Complimenting the findings from the previous sections, the trend suggests a scan–then–hold relationship: eye movements are varied on entry and rate of re-entry for some items (AOI- and layout-dependent), but dwell time per item after fixation is consistent across categories for a presentation of 10-s. Practically, this means that placement and visual salience (to influence TTFF/FC) could be more important than content category for dwell itself; moving actionable text and effectiveness cues to early-attention positions may thus reap greater benefits than efforts to prolong dwell by category.

#### 4.5.6. AOI-Level Fixation Duration Differences

For comparing time variation to fixation between AOIs in breast cancer screening advertisements, generalized linear mixed model analysis was conducted. A gamma distribution with a log link function was used to fit the distribution of time to fixation. It was positively skewed; thus, the ID of the participant as a random intercept was incorporated. Education Level, AOI levels, Age Group, and Household Type were included as fixed factors. All random slopes were excluded due to convergence issues and fixed variables in the random effects model. The analysis found a highly significant main effect of AOI on duration of fixations, χ^2^(40) = 176.566, *p* < 0.001, which meant the order of the participants’ fixation differed significantly depending on the specific visual feature (AOI) visible. Household Type, χ^2^(4) = 1.925, *p* = 0.750; Age Group, χ^2^(3) = 1.254, *p* = 0.740; and Education Level, χ^2^(3) = 0.525, *p* = 0.913, were not significant (Table 10).

The estimated marginal mean analysis also identified that there were certain AOIs whose fixation times were much higher than the grand mean. These were slogan text in Ad2_Text (EMM = 603.26 ms, Estimate = 0.774, SE = 0.110, *p* < 0.001), explanation callouts in Ad6_IconswithText (EMM = 376.95 ms, Estimate = 0.552, SE = 0.112, *p* < 0.001), symbolic ribbon in Ad3_Icon(ribbon) (EMM = 435.73 ms, Estimate = 0.448, SE = 0.118, *p* < 0.001), and slogan in Ad5_Text (EMM = 283.27 ms, Estimate = 0.303, SE = 0.112, *p* = 0.007). These factors did tend to accompany emotionally provocative stories or strongly visual material, which would indicate a willingness on the part of participants to devote more attentional resources to information that was informative or persuasion-based in nature. Alternatively, other AOIs were given very brief fixation times, such as the anatomical icon Ad2_Icon(breasts) (EMM = 178.26 ms, Estimate = –0.286, SE = 0.109, *p* = 0.009), the institutional logo of Ad6_YpourgioYgeias (EMM = 220.32 ms, Estimate = –0.234, SE = 0.109, *p* = 0.031), and the headlines of Ad5_HeadText (EMM = 236.15 ms, Estimate = –0.222, SE = 0.109, *p* = 0.041) and Ad4_HeadText (EMM = 209.18 ms, Estimate = –0.215, SE = 0.109, *p* = 0.048). These locations, occasionally featuring more formal institutional or historic signage, seemed to hold the audiences’ eyes for a briefer amount of time, perhaps resulting from lower emotional or perceived worth. AOIs with narrative/efficacy text and close-up (e.g., Ad2_Text, Ad6_IconswithText) or bear a conspicuous health symbol (Ad3_Icon(ribbon)) capture attention longer, approximately 1.3–2.2× the grand mean on the geometric scale. Anatomical icons, institutional logos, and certain headline headlines draw shorter processing after fixation. Together with §5.5.1 (revisit) and §5.5.2 (orient), this is in favor of a scan–then–read dynamic: viewers end up spending more dwell time on informative/narrative AOIs than on purely institutional or abstract cues.

In general, the findings show a distinct pattern, effectively engaging pictures and textually informative message components tended to sustain visual attention longer, and institutional or less polemical elements were granted briefer cognitive processing. This lends credence to the thesis that health promotion persuasive communication can be improved through the use of effectively engaging and visually informative messages.

## 5. Discussion

At the category level of resolution, the variations in attention were relatively small; symbols resulted in fewer fixation allocations, but TTFF and fractional dwelling (FD) did not differ significantly across broad categories of AOIs. However, there were relatively high levels of element-level (AOI) variation, suggesting that attention allocation is stimulated by particular instantiations rather than category membership in general (RQ1–RQ2). The demographic controls (age, education level, household income) were relatively unassociated with TTFF, fixation count (FC), or FD across both descriptive and linear mixed modeling analyses (GLMMs) (RQ3). Moreover, there were no meaningful or stable interactions across specifications between categories of AOIs and demographics (RQ4). Finally, those AOIs or other semantically relevant and efficacious elements (facing/efficacy panels, meaningful narrative text) stimulated greater allocations of dwelling time compared with logos or other cue-related information like source information, although logos/sources tended toward earlier attention allocation (shorter TTFF) but not dwelling (RQ5).

This research explored how particular design features in breast cancer screening ads direct visual attention in screening-eligible women (≥40 years). Three firm patterns were observed. Firstly, at the category level, the effects were modest; Fixation Count (FC) only trended by AOI category (Symbols below the grand mean), whereas Time to First Fixation (TTFF) and Fixation Duration (FD) did not systematically differ across wide categories. Second, at the AOI level, heterogeneity was salient. Some elements—especially narrative/efficacy text (e.g., Ad2_Text, Ad6_IconswithText) and the ribbon symbol in information-dense environments—engaged attention for longer periods, while institutional logos and anatomical icons were fixated briefly. TTFF revealed an “early orienters vs. late orienters” division; brief labels, logos, or source cues were tended to be fixated earlier, while big headlines and data blocks later, in line with a scan-then-read strategy. Third, demographic factors (household, education, age) were weak in zero-order tests and in combined models, indicating that design factors more than subgroup characteristics were the main determinants of attentional allocation in this sample.

### 5.1. Bivariate Relationships and Preliminary Trends

Early correlated contrast of eye-fixation measures provided the initial flicker of attentional behavior as a stimulus function. A single moderate positive correlation between Time to First Fixation and Fixation Count (ρ = 0.316, *p* < 0.001) confirms that fixated spots are longer to fixate at the next point in the viewing sequence and are more often fixated, as with more visual scrutiny or cognitive processing effort being expended on harder items. A less severe but more significant correlation between Fixation Duration and Fixation Count (ρ = 0.213, *p* < 0.001) indicates that objects that are frequently gazed at are also gazed at for a little longer, lending support to [24] argument, that count and duration are complementary measures of attention intensity.

Notably, the above correlations validate each measure’s theoretical distinctiveness, i.e., lack of correlation between TTFF and Fixation Duration (ρ = 0.040, *p* = 0.161), which is in support of the performance of research on them separately. These findings are in support of previous research by [14,17,64], wherein they emphasize the dynamic temporal character of attention as central to the understanding of message salience. Neuromarketing-wise, long-term pair and first fixation continues to be the preeminent method for measuring message resonance and cognitive elaboration [14,28].

### 5.2. Interpreting the Pattern: A Scan–Then–Read Pipeline

Our decoupling of when attention is engaged (TTFF) and how much it is engaged (FD), with minimal correlation between TTFF and FD and modest coupling of FC with each, is consistent with bounded-capacity accounts and temporal division of labor in dual-process accounts. Xiu et al. [19] demonstrated that peripheral cues enable early attentional capture (N1), while central, argument-valid content monopolizes later cognitive resources (P3). Our findings reflect that rhythm, early orienters are concise cues (logos, brief labels, some symbols), but more elaborative processing is the job of efficacy/narrative text blocks after gaze arrival. Bullock et al. [20] contend that narratives persuade through processing fluency, which is useful in explaining longer dwellings on slogans and brief efficacy copy; these materials are readable, understandable, and map directly onto action. Lam et al. [21] also discovered that infographics (well-organized visuals + text) support elaboration when compared to text-based messages by themselves, arguing the case that visual organization is not peripheral but fundamental to thought.

More significantly, null category effects in combination with substantial AOI-level differences reveal that attention is less semantically class-guided in the abstract (“text,” “image,” “logo”) than guided by the manner in which particular instances are realized in layout—their size, distance to the central beginning, local contrast, and co-appearance with actionable claims. This result is meeting Klein and colleagues’ discovery that image–text congruence converts attention into knowledge; consumers spend additional look time whenever what they first glance at naturally bridges what they subsequently must learn. In our stimuli, slogans on our stories and brief efficacy messages do prove to be just that bridge, earning revisits and stick once seen—even when they are technically not the first things gazed at [32,37].

### 5.3. Emotion, Threat, and Efficacy: Routing Arousal Toward Action

One of the long-standing issues is whether prevention messages can use appeals to emotion. Brigaud et al. [17] demonstrated that humor inhibits avoidance and maintains attention, whilst Avery et al. [18] discovered that fear imagery saw attention predict stronger vaccination intention, while attention to non-fear imagery could actually harm recall. Our findings are consistent with the general agreement in EPPM-oriented research [26,37], efficiency needs to be combined with arousal to convert attention into adaptive influence. We observed that two affect-based features (e.g., a heart symbol in a home advertisement) received very late fixations and, when late, did not sustain dwell unless assisted by overt verbal affirmation. Conversely, AOIs that combined symbolic cues with action-oriented text (e.g., icons-with-text callouts) created longer dwell—a pattern consistent with channeling initial arousal into a productive decision stream [37].

The exclusion from consideration in our AOI-level models of institutional logos and certain anatomical symbols is in line with criticisms of pink-ribbon cause marketing [29,30], where cues by symbolism surpass explicit guidance. Our results fill the gaps: symbols and logos can perform as first-order orienters when small and positioned centrally, but they begin to degrade alone; in order to function as persuaders at all, they ought to be collocated with access information (telephone numbers, booking links) or efficacy statements. This is supported by warning-label studies [24], in which size and colour design features manipulate attention and memory, and revelation markers draw differential attention—micro-cues can be effective [23,25], but location relative to the CTA makes them useful.

### 5.4. Audience Considerations: Older Adults, Literacy, and Cultural Cues

Although demographic main effects were mostly null here, the wider literature implies key design accommodations. Yılmaz et al. [28] demonstrated that health literacy influences the amount of time women look at complicated displays; older patients might exhibit longer per-fixation dwell on content they consider relevant. Wang et al. [14] demonstrated that prevention-oriented, safety-framed images engage more attention and intention in older adults than threat-filled designs. Lumpkins’s [40] study with African American women showed that culturally specific symbols (e.g., the Christian cross) can serve as central meaning cues instead of peripherals, enhancing ad attitudes across levels of involvement. Placed in conjunction with our AOI results, these findings imply that category labels hide critical within-category tailoring, type size, contrast, hierarchical salience, and cultural relevance, likely affecting whether “Text” or “Symbol” operates as a central route cue or a disregarded decoration.

These results combined address RQ2 by demonstrating a systematic AOI type difference in gaze behavior, and RQ4 by demonstrating that said difference is content-driven to a significant degree and not moderately moderated by demographic background. They provide a qualified solution to RQ1 in as much as they demonstrate emotionally engaging and textually rich content to be more engaging and attention-grabbing than symbolic or institutional information.

The research contributes to three dimensions. One, they establish that attention is multi-component, with orientation latency (TTFF), revisitation (FC), and processing depth (FD) being distinct but related. Fixing “attention” as a single construct makes design levers fuzzy. Two, the AOI-over-category result is reconcilable with a layout-sensitive elaboration account; a semantic class may be peripheral or central based on realization (size, position, congruence). Third, the findings are a match for an EPPM/ELM hybrid, peripherally orienting (logos, small icons) is sufficient to open the gate, but central, fluent, efficacy-loaded text seems to be required to sustain attention in ways plausibly likely to lead to informed action—closing the chronic gap between arousal and uptake of the screening literature (Table 11).

## 6. Practical Implications

This research aimed to find out what elements of breast cancer screening messages are most likely to attract and sustain visual attention in women aged forty years and older. The findings show moderate variation at the level of broad categories but radical variation at the level of individual elements, and a scan–then–read pattern whereby short cues receive early fixations and narrative and efficacy text sustains attention once entered. Demographic influences were negligible, indicating that message design decisions, more so than audience segmentation, represent the key levers for heightening attentional allocation. The findings have a number of direct practical implications for policy, communication practice, and education that address the study’s aim of informing message design with the potential to enhance screening uptake.

### 6.1. Policy and Public Health Communication

For public health program administrators and policymakers, the immediate implication is to make “action adjacency” a design principle. Indicators of trustworthiness, such as badges or logos, must be colocated with explicit next steps, such as instructions for reservation, price display, and temporal expectations, within the same visual unit [32,33]. The study reveals that logos are not necessarily effective on their own; their persuasive power is enhanced when visually associated with a clear avenue for action. Public programs can make this feasible by providing standardized, plain templates for posters, invitation letters, SMS landing pages, and social tiles, putting the brief narrative slogan, the efficacy micro-message, and the call-to-action in early-attention positions (e.g., center or top left), keeping crowded statistics back for a secondary layer. Those templates would have to specify minimum type sizes, contrast levels, and maximum line lengths appropriate for mid-life and older readers, and have to include plain-English equivalents to reduce text densities without sacrificing content. With the importance of early attention, procurement procedures should require pre-flight attention testings—be it with small-sample eye-tracking or vetted click/scroll heat methods—with passing requirements on time-to-CTA and proportion of viewers who lock into call-to-action and source within the first few seconds of view. Because the research discovered that few demographic main effects existed, equity can be promoted by universal design as a foundation, augmented by low-literacy and language variation, and by targeted channel and messenger choice rather than convergent core designs [28,39]. Measurement should also reflect attention measures, time-to-click measurement, booking widget engagement, and completion rates, offering a cycle of feedback to optimize iteratively during campaign flight.

### 6.2. Advertising Strategy and Creative Direction

Health teachers, patient advocates, and community organizations can use these findings and convert them into activities and materials that demonstrate the intended behavior. Slides and handouts must sit booking steps and eligibility requirements next to the slogan and familiar source, with QR codes linking to short, mobile-specific forms. Workshops need live scheduling demonstrations so learners may walk through the flow when attention is already high. Since research and earlier studies have proven that image-text congruency facilitates learning, the pictures showing the modeled behavior must then be followed by exactly equivalent instructions [29,35]. Where culturally evocative symbols are to be used, they must be placed just next to the call-to-action and efficacy statements so that they can act as carriers of meaning and not as superficial decoration. For less literate target audiences, “one-idea” tiles—a mix of a brief slogan and one action in large high-contrast letters are best suited to interpersonal sharing modes like messaging or local pages [36,40].

Creative and media agencies must include routing outputs and layout diagnostics as part of regular optimization. Prior to spending commitment, call-to-action block size and skew must be monitored to place it in locations that are likely to attract initial fixations, and media format must be selected in order to guarantee advantage (e.g., ongoing eye-level poster placement or top-view mobile supply). Optimization must target to drive time-to-CTA and CTA fixation levels alongside more traditional recall or liking metrics, as minimal adjustments to proximity and order, stacking the call-to-action above the headline, can significantly boost revisitation and dwell on effective content. Concurrently, message frames should not be perceptually noisy; there should be an unambiguous route from slogan to efficacy cue to call-to-action that minimizes competition between items and is consistent with the scan–then–read paradigm embodied in the data.

### 6.3. Healthcare Providers, Hospitals, and CSR Partners

Health care practitioners, hospitals, and corporate social responsibility sponsors may use the same principles to co-branded outreach [36,41]. The sponsor or hospital logo works best in co-branding when it is functionally coupled with the booking continuum—by being paired with a telephone number, QR code, or scheduling link—so that credibility and action are processed simultaneously. Wherever there is space to insert eligibility and “what to expect” micro-copy, put it to the right of the booking button, backed up by short narrative leads (e.g., “Most women take fifteen minutes”) to exploit longer dwell time for shorter efficacy copy. Staff attending to patients need to be instructed to start with next actions and tangible payoffs the moment after any emotional foreplay, verbal scripts consistent with the visual sequence assist in aligning attention for the first time with tangible action. For corporate campaigns, cause marketing accountability in responsible cause marketing entails a minimum amount of information content within the main leading visual block; symbol-alone creative should only be utilized if an action prompt is found in the same location in the configuration [18,24,25].

### 6.4. Cross-Sector Collaboration

Regulators and funders can assist such practice by implementing readability and action guidelines for publicly funded screening communications. Outlining recommendations for minimum font size, contrast, and co-location of source and call-to-action would provide vendors and grantees with a consistent standard to follow. The inclusion of evaluation terms in grants or contracts can mandate pre-flight review and post-flight reporting of process measures rather than gross reach or impressions. Where testing includes the application of eye-tracking, arrangements for consent and data minimization should be laid down; where this is impracticable, proxy measures that have been validated are an acceptable fall-back.

Together, these effects impose an easy-to-apply working rule that honors both the results of the study and general evidence, sets up the scan–then–read pipeline so that eyes first look at trustworthy, actionable information for an initial interval, and then only long enough to read smooth, efficacy-generating text to inform the next action. Policies, communications, and education interventions using this pipeline through channels are likely to yield availability of screening leading to participation, and not relying on heavy segmentation or fear appeals that the evidence indicates are less effective in the target age group.

## 7. Conclusions, Limitations, and Future Directions

This research investigated how specific elements of breast cancer screening messages direct visual attention in women aged forty and above. Based on AOI-level eye-tracking data and mixed-effects models, we concluded that there were small category-level differences but high heterogeneity on an individual level. Brief cues and source points were more easily encountered initially, but brief narrative and efficacy text kept attention while accessed, logos and abstract signs seldom held dwell unless visually accompanied by an explicit path to action. Demographic trends were otherwise low-keyed, lending support to the conclusion that layout and realization, rather than audience segmentation in itself, most significantly influenced attention. When taken cumulatively, the findings corroborate a scan–then–read process; initial orienters must be designed to guide readers toward fluid, effectiveness-packed copy located near prominent calls-to-action.

A number of productive avenues for research can build upon and expand these results. The findings are exploratory and bounded by the six-stimulus, static-image design and a screening-eligible sample; external validity should be assessed through replication with larger, more diverse creatives (including letters/SMS/landing pages) and channels. One obvious next step is to increase the design without sacrificing experimental control. Multi-site, preregistered studies with larger, stratified samples would allow for by-participant random slopes and more stable estimation of individual differences, while also assessing whether the scan–then–read pattern replicates across regions and delivery modes. Expanding the array of stimuli from six static advertisements to dynamic and interactive types—brief videos, carousels, and SMS landing pages—would come closer to real media environments and enable researchers to investigate if motion, sound, and progressive disclosure change orientation and dwell [20,24,26].

Orienting temporal dynamics deserves more precise scrutiny. Experiments in the future can be controlled for initial fixation and floors of latency by employing off-center commencement, drift correction, and pre-specification of physiologically realistic minima for TTFF. Survival models with unfixed areas as right-censored events offer a principled environment to handle latency data; a log-normal AFT model comparison with gamma/log-link specifications would elucidate robustness. Since revisits are usually over-dispersed, counts of fixations should be the default to use negative binomial GLMMs with log links. Conditioning on AOI area and eccentricity, and face presence and text density where necessary, as covariates will remove content from size and position biases. Hierarchical or Bayesian partial pooling can control multiplicity when multiple AOIs are being handled, while retaining interpretable, regularized estimates for single items [21,64]. Beyond the AOI-level statistics reported, future work will provide participant-aggregated heatmaps (and subgroup panels) using standardized kernels and normalization, to visually triangulate the GLMM findings without overloading the current article. We analyzed neither the post-exposure rating data nor the facial-expression (FEA) data in this research and concentrated only on gaze parameters measured at the area-of-interest (AOI) level and inferential modeling. The additional data would be discussed in another report in order not to overburden this current write-up.

Closing the attention-to-behavior gap remains the most critical one. A/B testing in the field can balance on a limited number of design knobs—logo and CTA co-location, narrative slogan near efficacy micro-copy distance, type size and contrast—and process metrics approximating eye-tracking (time-to-CTA, CTA fixation surrogates like first-click or scroll heat, dwell-time on booking blocks), along with outcomes further down the funnel, click-through, booking, and attendance [21,65]. Combining these trials with low-burden understanding tests, decisional conflict questionnaires, and expected regret scales would elucidate whether attention to efficacy information leads to informed choice and adoption.

Arousal-action balance of content also requires systematic testing. Sequential designs can be used to manipulate the timing and distance of efficacy and emotion cues to test dose–response relationships; for example, whether presenting the “how to book” message within the same visual container as an affective image leads to quicker and longer CTA interaction, or how these vary by age bands or by literacy. The second productive axis is connecting cultural endorsements and symbols. Such tests that place symbols pertinent to the group side by side with access information can determine if symbols act as core meaning cues in a sub-group fashion or not [6,7].

Equity is supported by design, rather than burdensome segmentation. Experimental control over typography, contrast, and text density by literacy level can define thresholds that support dwell without burden. Since our findings demonstrate that layout consistently outperforms demographics in predicting attention, subsequent research can compare “universal” templates, large text, high contrast, plain hierarchy, to more segmented competitors to identify the cost-effective route to inclusive effectiveness. Cross-national research would provide external validity by examining whether program designs, invitation systems, cost framing, and access channels are interactive with design in influencing attention and behavior in countries other than Greece [3].

Lastly, field-to-lab methodological convergence will reinforce causal arguments. Hybrid methods blending short lab-based eye-tracking with large-scale online deployments via validated attention proxies can speed up iteration. Wherever budget permits, multimodal measures—facial expression analysis, pupillometry, or lightweight EEG—can be included to determine if arousal moments are being channeled toward efficacy and action, as these findings suggest. Transparency in preregistration, open AOI annotations, and shared stimulus repositories will allow for cumulation and comparability between studies. As a next step, AI-assisted prototyping of creatives that implement these rules could be evaluated against human-designed variants in a controlled eye-tracking study; such generative simulation is beyond the scope of the present analysis.

Overall, what our research describes is a humble choreography, let responsible, actionable information be the first thing the eye alights upon, and let straightforward, sinuous direction keep it in view for just long enough to have impact. If there is a romance of public health, it is in such humble decisions—the logo branded onto a lifeline, the headline that softly invites the reader onto a booking path, the type that summons rather than yaps. What is left is to perform this score at scale and in many places, until there is a regular beat: from glance to grasp, from arousal to effect, from availability to presence. When messages respect this trajectory, care emerges out of attention, and a moment on the page is a step toward a life resumed.

## Figures and Tables

**Table 1:** Sample profile.

Characteristic		N	Percentage
Age group	40–45	9	30.0%
	46–50	5	16.7%
	51–55	7	23.3%
	56–60	9	30.0%
Household type	Married with children	19	63.3%
	Married without children	3	10.0%
	Single with children	3	10.0%
	Single without children	5	16.7%
Education	Primary school	4	13.3%
	Compulsory high school	13	43.3%
	University	7	23.3%
	Master’s degree and above	6	20.0%

**Table 2:** AOI descriptions and Examples.

AOI Category	Description	Examples
**Text**	Headlines, body text, or captions	HeadText, SmallText1, IconswithText
**Symbol**	Ribbons, hearts, symbolic elements (e.g., breast/statistics icons)	Icon (ribbon), Icon (heart), Icon1Statistics
**Image/Visual**	Photographs, illustrations, or drawings	Woman’s Face, Hand-drawn Figure, Magnifier
**Logo**	Corporate or organizational logos	Alpha Bank, ProtoThema, Ygeia
**Website**	Stylized URLs or web references	www.alpha.gr, www.hygeia.gr
**Source/Authority**	Institutions, endorsing bodies, or political figures	Fofi Gennimata, Ministry of Health, Government

**Table 3:** Descriptive Statistics.

Statistic	Household	Age	Education	Fixation Count	TTFF (AOI)	Fixation Duration
Mean	1.833	2.533	2.167	1.340	641.285	289.162
Std. Deviation	1.241	1.204	1.214	1.280	1644.718	449.974
Interquartile Range (IQR)	2.000	3.000	2.000	2.000	343.817	118.570
Skewness	1.158	−0.078	0.463	1.050	3.562	12.195
Std. Error of Skewness	0.070	0.070	0.070	0.070	0.070	0.070
Kurtosis	−0.132	−1.538	−1.384	0.980	13.014	199.948
Std. Error of Kurtosis	0.139	0.139	0.139	0.139	0.139	0.139
Shapiro–Wilk	0.688	0.824	0.786	0.861	0.435	0.276
*p*-value (Shapiro-Wilk)	<0.001	<0.001	<0.001	<0.001	<0.001	<0.001

**Table 4:** Spearman correlations among gaze metrics and demographics.

Variables	Spearman’s ρ	*p*-Value	95% CI (Lower)	95% CI (Upper)
Fixation Count—TTFF	0.316	<0.001	0.264	0.365
Fixation Count—Fixation Duration	0.213	<0.001	0.159	0.266
Fixation Count—Household	0.004	0.892	−0.052	0.060
Fixation Count—Age	−0.003	0.930	−0.058	0.053
Fixation Count—Education	0.022	0.436	−0.034	0.078
TTFF—Fixation Duration	0.040	0.161	−0.016	0.096
TTFF—Household	−0.007	0.812	−0.063	0.049
TTFF—Age	−0.035	0.221	–0.091	0.021
TTFF—Education	–0.038	0.184	−0.094	0.018
Fixation Duration—Household	0.024	0.406	−0.032	0.080
Fixation Duration—Age	−0.010	0.717	−0.066	0.046
Fixation Duration—Education	−0.015	0.605	−0.071	0.041
Household—Age	−0.366	<0.001	−0.413	−0.316
Household—Education	0.151	<0.001	0.096	0.205
Age—Education	−0.082	0.004	−0.138	−0.027

**Table 5:** Generalized Linear Mixed Model (GLMM) results for fixation count across AOI categories.

Effect/Term	df	χ^2^	t	Estimate	SE	*p*	M (Fixations)	95% CI Lower	95% CI Upper	Interpretation
ANOVA (Type III, Wald χ^2^)										
Intercept	1	203.813	–	–	–	<0.001	–	–	–	–
AOI category	5	9.958	–	–	–	0.076	–	–	–	–
Household	4	4.109	–	–	–	0.391	–	–	–	–
Age	3	0.599	–	–	–	0.897	–	–	–	–
Education	3	2.458	–	–	–	0.483	–	–	–	–
Fixed Effects (log scale)										
Intercept	–	–	131.466	7.714	0.059	<0.001	–	–	–	–
Logo	–	–	1.844	0.141	0.077	0.065	–	–	–	–
Source/Authority	–	–	−1.086	−0.110	0.101	0.278	–	–	–	–
Symbol *	–	–	−2.283	−0.164	0.072	0.022	–	–	–	Significantly fewer fixations
Text	–	–	−0.485	−0.029	0.061	0.628	–	–	–	–
Website	–	–	1.537	0.088	0.057	0.124	–	–	–	–
Estimated Marginal Means (response scale)										
Image	–	–	–	–	243.82	–	2579.21	2142.99	3104.23	Highest attention overall
Text	–	–	–	–	176.61	–	2445.43	2122.66	2817.29	Mid-range attention
Website	–	–	–	–	315.38	–	2413.29	1867.97	3117.81	Comparable to Text
Symbol *	–	–	–	–	170.33	–	2174.73	1865.24	2535.56	Significantly fewer fixations
Logo	–	–	–	–	248.09	–	2006.36	1574.56	2556.59	Lower attention
Source/Authority	–	–	–	–	167.94	–	1900.14	1597.92	2259.52	Lowest attention

Note. Results are based on the GLMM with a gamma distribution and a log link. Estimates are marginal means on the response scale. * *p* < 0.05 (significantly different from grand mean).

**Table 6:** Generalized Linear Model (GLM) results for fixation count across individual AOIs.

Effect/AOI Label	df	χ^2^	Estimate	*p*	EMM (Fixations)	Interpretation
Omnibus Tests (Type III, Wald χ^2^)						
Intercept	1	205.31	–	<0.001	–	Model baseline significant
AOI (40 levels)	40	99.79	–	<0.001	–	Strong AOI effect
Household	4	3.85	–	0.427	–	Not significant
Age	3	0.18	–	0.981	–	Not significant
Education	3	4.00	–	0.262	–	Not significant
Selected AOI EMMs and GLM Estimates						
Ad2_Icon (heart)	–	–	+0.654	<0.001	4227.95	Significantly higher FC
Ad1_HeadText	–	–	+0.464	0.009	3498.05	Significantly higher FC
Ad4_HeadText	–	–	+0.431	0.016	3302.05	Significantly higher FC
Ad4_Text	–	–	+0.406	0.023	3384.90	Significantly higher FC
Ad6_FofiGenimata	–	–	−0.683	<0.001	1589.28	Significantly lower FC
Ad4_Icon (breast)	–	–	−0.613	<0.001	1110.44	Significantly lower FC
Ad5_HeadText	–	–	−0.544	0.002	1276.46	Significantly lower FC
Ad2_Kivernisi	–	–	−0.396	0.028	1480.82	Lower FC

Note. Model fitted with a gamma distribution and a log link. Participant ID included as a random intercept. Random slopes for Household, Age, and Education were removed due to a lack of within-group variance. Model singularity may affect the reliability of estimates. Type III sum of squares is used for testing fixed effects.

**Table 7:** Generalized Linear Model (GLM) results for Time to First Fixation (TTFF) by AOI category.

Effect/AOI Category	χ^2^	*p*	EMM (ms)	GLM Estimate	Interpretation
Omnibus Tests					
Intercept	121.212	<0.001	–	–	Significant—confirms model convergence, not substantive
AOI_category	0.000	1.000	–	–	No effect—TTFF did not differ across AOI categories
Household	0.954	0.917	–	–	No effect
Age	5.598	0.133	–	–	Not significant
Education	0.000	1.000	–	–	No effect
AOI Category Estimates					
Website	–	–	291.32	–	Fastest initial fixation
Source/Authority	–	–	343.84	–	Quick to attract gaze
Image	–	0.549	377.25	–0.159	Early orienting (near sources/CTAs)
Logo	–	0.024	443.44	0.385	Slower than sources/CTAs/images
Text	–	0.006	650.23	0.442	Significantly slower initial fixation, late orienting (reading requirement)
Symbol	–	0.101	688.56	–0.252	Latest orienting (abstract iconography)

Note. GLM estimates are log-transformed deviations from the grand mean under sum contrast coding. EMMs are back-transformed to the original TTFF scale. Estimates for Website and Source/Authority are implicitly included in the reference level.

**Table 8:** Generalized Linear Model (GLM) results for Time to First Fixation (TTFF) across individual AOIs.

Effect/AOI Label	df	χ^2^	*p*-Value	EMM (ms)	GLM Estimate	Interpretation
Omnibus Tests (Type III, Wald χ^2^)						
Intercept	1	119.426	<0.001	–	–	The model has a strong intercept (baseline TTFF)
AOI (40 levels)	40	268.137	<0.001	–	–	Statistically significant effect of AOI on TTFF
Household	4	0.346	0.987	–	–	NS—no effect of household status
Age	3	1.133	0.769	–	–	NS—no age effect
Education	3	3.266	0.352	–	–	NS—no education effect
AOI-Level Estimates						
Ad6_Headtext	–	–	<0.001	52.83	–2.714	Fastest gaze onset
Ad2_FofiGenimata	–	–	<0.001	30.65	–2.546	Extremely early fixation
Ad3_Icon (ribbon)	–	–	<0.001	25.91	–1.268	Very early fixation
Ad4_Picture (womanhand-drawn)	–	–	0.016	108.59	–0.793	Early fixation
Ad3_BusinessLogo(Ygeia)	–	–	0.009	151.18	–0.950	Significantly faster than average
Ad2_Icon (heart)	–	–	<0.001	1201.38	+1.621	Slowest gaze onset
Ad1_HeadText	–	–	<0.001	1268.18	+1.317	Very slow initial fixation
Ad3_Icon3Statistics	–	–	<0.001	1459.11	+1.214	Delayed attention
Ad3_SmallText3	–	–	<0.001	1316.80	+1.143	Delayed attention
Ad6_picture (fist)	–	–	<0.001	1232.67	+1.123	Delayed attention
Ad3_SmallText1	–	–	<0.001	1226.52	+1.177	Delayed attention
Ad4_HeadText	–	–	0.005	1976.92	+0.924	Very delayed gaze entry

Note. GLM estimates are on the log-transformed scale; positive values reflect slower fixation onset compared to the grand mean. EMMs represent actual TTFF in milliseconds.

**Table 9:** Generalized Linear Mixed Model (GLMM) results for Fixation Duration (FD) across AOI categories.

Effect/AOI Category	df	χ2	*p*-Value	EMM (ms)	GLM Estimate	Interpretation
Omnibus Tests (Type III, Wald χ^2^)						
Intercept	1	153.739	<0.001	–	–	The model baseline is significant
AOI category	5	7.355	0.196	–	–	n.s.: FD does not vary by category
Household	4	5.752	0.218	–	–	n.s.
Age	3	1.866	0.601	–	–	n.s.
Education	3	1.473	0.689	–	–	n.s.
Estimated Marginal Means (EMMs; ms, descriptive)						
Website	–	–	0.225	251.47	~–0.074	Shortest fixation duration (NS)
Image	–	–	0.485	253.71	~–0.046	Short fixation duration (NS)
Logo	–	–	0.089	260.79	~+0.084	Marginally longer fixation duration
Symbol	–	–	0.124	271.99	~+0.123	Slightly longer, not significant
Text	–	–	0.955	297.17	~–0.004	Mid-range fixation duration (NS)
Source/Authority	–	–	—	308.82	–	Highest fixation duration (baseline)

Note. Results are based on the GLMM with a gamma distribution and a log link. EMMs represent back-transformed estimates on the response scale.

**Table 10:** Generalized Linear Mixed Model (GLMM) results for Fixation Duration (FD) across AOIs.

Effect/AOI Label	df	χ^2^/z	*p*-Value	EMM (ms)	Estimate (log)	Ratio vs. Grand Mean	Interpretation
Omnibus Tests (Type III, Wald χ^2^)							
Intercept	1	144.370	<0.001	–	–	–	Model baseline significant
AOI (40 levels)	40	176.566	<0.001	–	–	–	Significant differences in FD
Household	4	1.925	0.750	–	–	–	Not significant
Age	3	1.254	0.740	–	–	–	Not significant
Education	3	0.525	0.913	–	–	–	Not significant
Selected AOIs (EMMs and coefficients)							
Ad2_Text	–	7.032	<0.001	603.26	+0.774	≈2.17×	Much longer dwell
Ad6_IconswithText	–	4.937	<0.001	376.95	+0.552	≈1.74×	Longer dwell
Ad3_Icon (ribbon)	–	3.801	<0.001	435.73	+0.448	≈1.57×	Longer dwell
Ad5_Text	–	2.706	0.007	283.27	+0.303	≈1.35×	Longer dwell
Ad2_Icon (breasts)	–	–2.629	0.009	178.26	–0.286	≈0.75×	Shorter dwell
Ad6_YpourgioYgeias	–	–2.151	0.031	220.32	–0.234	≈0.79×	Shorter dwell
Ad5_HeadText	–	–2.046	0.041	236.15	–0.222	≈0.80×	Shorter dwell
Ad4_HeadText	–	–1.981	0.048	209.18	–0.215	≈0.81×	Shorter dwell

Note. GLMM fitted with a gamma distribution and a log link; Participant ID included as a random intercept. EMMs are back-transformed fixation durations (ms). Ratios are multiplicative differences relative to the grand mean.

**Table 11:** Summary table of AOI-level gaze patterns across metrics.

AOI Label	FC	TTFF	FD	One-Line Takeaway
Ad2_Icon (heart)	↑↑	↑↑ slow	—	Emotional icon draws many looks but is late to attract the first gaze.
Ad1_HeadText	↑	↑↑ very slow	—	A big headline gets many fixations once reached.
Ad4_HeadText	↑	↑↑ very slow	↓	Headline looked at often, entered late, processed briefly.
Ad4_Text	↑	—	—	Body copy engages frequent revisits.
Ad6_FofiGenimata	↓↓	—	—	Institutional name receives few fixations.
Ad4_Icon (breast)	↓↓	—	—	An anatomical icon draws minimal looks.
Ad5_HeadText	↓	—	↓	The header attracts fewer fixations and brief processing.
Ad2_Kivernisi	↓	—	—	Government cue is under-attended.
Ad6_Headtext	—	↓↓ fastest	—	The header was rapidly spotted.
Ad2_FofiGenimata	—	↓↓ extremely fast	—	The source cue is seen almost immediately.
Ad3_Icon (ribbon)	—	↓ very early	↑	Ribbon grabs attention early and holds it.
Ad4_Picture (womanhand-drawn)	—	↓ early	—	Self-exam figure is an early orienter.
Ad3_BusinessLogo (Ygeia)	—	↓ faster than avg	—	The hospital logo is quickly noticed.
Ad3_Icon3Statistics	—	↑ delayed	—	Stats icon draws late attention.
Ad3_SmallText3	—	↑ delayed	—	Dense text entered late.
Ad6_picture (fist)	—	↑ delayed	—	First image noticed later.
Ad3_SmallText1	—	↑ delayed	—	Dense text entered late.
Ad2_Text	—	—	↑↑	Slogan/efficacy copy sustains longest dwell.
Ad6_IconswithText	—	—	↑	Icons with explanatory text hold attention.
Ad5_Text	—	—	↑	Copy retains processing time.
Ad2_Icon (breasts)	—	—	↓	Anatomical icon processed briefly.
Ad6_YpourgioYgeias	—	—	↓	Ministry logo receives short dwell.

Note. FC/FD: ↑ higher than grand mean; ↓ lower than grand mean (↑↑/↓↓ = stronger deviation). TTFF: ↓ faster onset; ↑ slower onset (↓↓ = fastest; ↑↑ = slowest). “—” = not part of the reported “selected AOIs” for that metric.

## Data Availability

The data presented in this study are available on request from the corresponding author.

## Abbreviations

The following abbreviations are used in this manuscript:

AOI	Area of Interest
ANOVA	Analysis of Variance
CI	Confidence Interval
CTA	Call to Action
ELM	Elaboration Likelihood Model
EMM (s)	Estimated Marginal Mean(s)

## Appendix A

**Table A1:** AOIs and AOI categories per screening advertisement.

Ad	Area of Interest (AOI)	AOI Category
Ad1	WomansFace	Image
	Icon (ribbon)	Symbol
	HeadText	Text
	Icon (heart)	Symbol
Ad2	Icon (heart)	Symbol
	HeadText	Text
	Family	Image
	Icon (breasts)	Symbol
	Text	Text
	FofiGenimata	Source/Authority
	YpourgioYgeias	Source/Authority
	Kivernisi	Source/Authority
Ad3	HeadText	Text
	Icon (ribbon)	Symbol
	Icon1Statistics	Symbol
	SmallText1	Text
	Icon2Statistics	Symbol
	SmallText2	Text
	Icon3Statistics	Symbol
	SmallText3	Text
	BusinessWesbsite (Ygeia)	Source/Authority
	BusinessLogo (Ygeia)	Logo
Ad4	Picture (womanhand-drawn)	Image
	HeadText	Text
	Icon (breast)	Symbol
	Text	Text
	BusinessWebsite (AlphaBank)	Website
	BusinessLogo (AlphaBank)	Logo
Ad5	HeadText	Text
	Picture (magnifier/breasts)	Image
	Text	Text
	BusinessWebsite (ProtoTheme)	Website
	BusinessLogo (ProtoThema)	Logo
Ad6	Headtext	Text
	Icon (ribbon)	Symbol
	picture(fist)	Image
	IconswithText	Text
	Website	Website
	FofiGenimata	Source/Authority
	YpourgioYgeias	Source/Authority
	Kivernisi	Source/Authority

**Table A2:** Kruskal–Wallis test and Dunn’s post hoc comparisons for fixation duration across household types.

Factor	χ^2^ (df)	*p*		Rank ε^2^ (95% CI)			
Household	9.762 (4)	0.045		0.008 [0.003, 0.026]			
Pairwise Comparison	z	W_i_	Wⱼ	rᵣb	*p*	*p* (Bonf.)	*p* (Holm)
MarriedWithKids—MarriedWithoutKids	−0.784	605.649	632.683	0.044	0.433	1.000	1.000
MarriedWithKids—SingleWithKids	−2.545	605.649	693.333	0.143	0.011	0.109	0.098
MarriedWithKids—SingleWithOutKids	−0.304	605.649	614.918	0.015	0.761	1.000	1.000
MarriedWithKids—SingleWithoutKids	1.506	605.649	519.951	0.140	0.132	1.000	0.754
MarriedWithoutKids—SingleWithKids	−1.339	632.683	693.333	0.101	0.181	1.000	0.754
MarriedWithoutKids—SingleWithOutKids	0.419	632.683	614.918	0.027	0.675	1.000	1.000
MarriedWithoutKids—SingleWithoutKids	1.760	632.683	519.951	0.188	0.078	0.784	0.549
SingleWithKids—SingleWithOutKids	1.851	693.333	614.918	0.124	0.064	0.642	0.513
SingleWithKids—SingleWithoutKids	2.707	693.333	519.951	0.289	0.007	0.068	0.068
SingleWithOutKids—SingleWithoutKids	1.531	614.918	519.951	0.143	0.126	1.000	0.754

Note. χ^2^ = Kruskal–Wallis test statistic. rᵣb = rank-biserial correlation effect size. Holm- and Bonferroni-corrected *p*-values are reported.

**Table A3:** Kruskal–Wallis test and Dunn’s post hoc comparisons for fixation duration across age groups.

Factor	Statistic	df	*p*	Rank ε^2^ (95% CI)			
Age	6.809	3	0.078	0.006 [0.001, 0.019]			
Pairwise Comparison	z	W_i_	Wⱼ	rᵣb	*p*	*p* (Bonf.)	*p* (Holm)
46–50 vs. 51–55	–2.403	575.278	653.312	0.125	0.016	0.098	0.099
51–55 vs. 56–60	1.911	653.312	599.883	0.085	0.056	0.336	0.280

Note. χ^2^ = Kruskal–Wallis test statistic. rᵣb = rank-biserial correlation effect size. Holm- and Bonferroni-corrected *p*-values are reported.

**Table A4:** Kruskal–Wallis test and Dunn’s post hoc comparisons for fixation duration across education levels.

Factor	χ^2^ (df)		*p*		Rank ε^2^ (95% CI)		
Education	6.667 (3)		0.083		0.005 [0.0007, 0.019]		
Pairwise Comparison	z	W_i_	Wⱼ	rᵣb	*p*	*p* (Bonf.)	*p* (Holm)
Master’s Degree and Above—Primary School	2.522	658.85	568.54	0.140	0.012	0.070	0.070

Note. Only significant or near-significant contrasts are shown. rᵣb = rank-biserial correlation.
